# Supplementary material for: The Set1 N-terminal domain and Swd2 interact with RNA polymerase II CTD to recruit COMPASS
Source: Nat Commun. 2020 May 1;11:2181. doi: 10.1038/s41467-020-16082-2 (PMC7195483; doi:10.1038/s41467-020-16082-2)
Supplement: Supplementary file 1 — Supplementary Information [file 41467_2020_16082_MOESM1_ESM.pdf]

Supplementary Information for Bae, Dubarry et al.

The Set1 N-terminal domain and Swd2 interact with RNA polymerase II CTD to recruit COMPASS

Supplementary Fig 1.

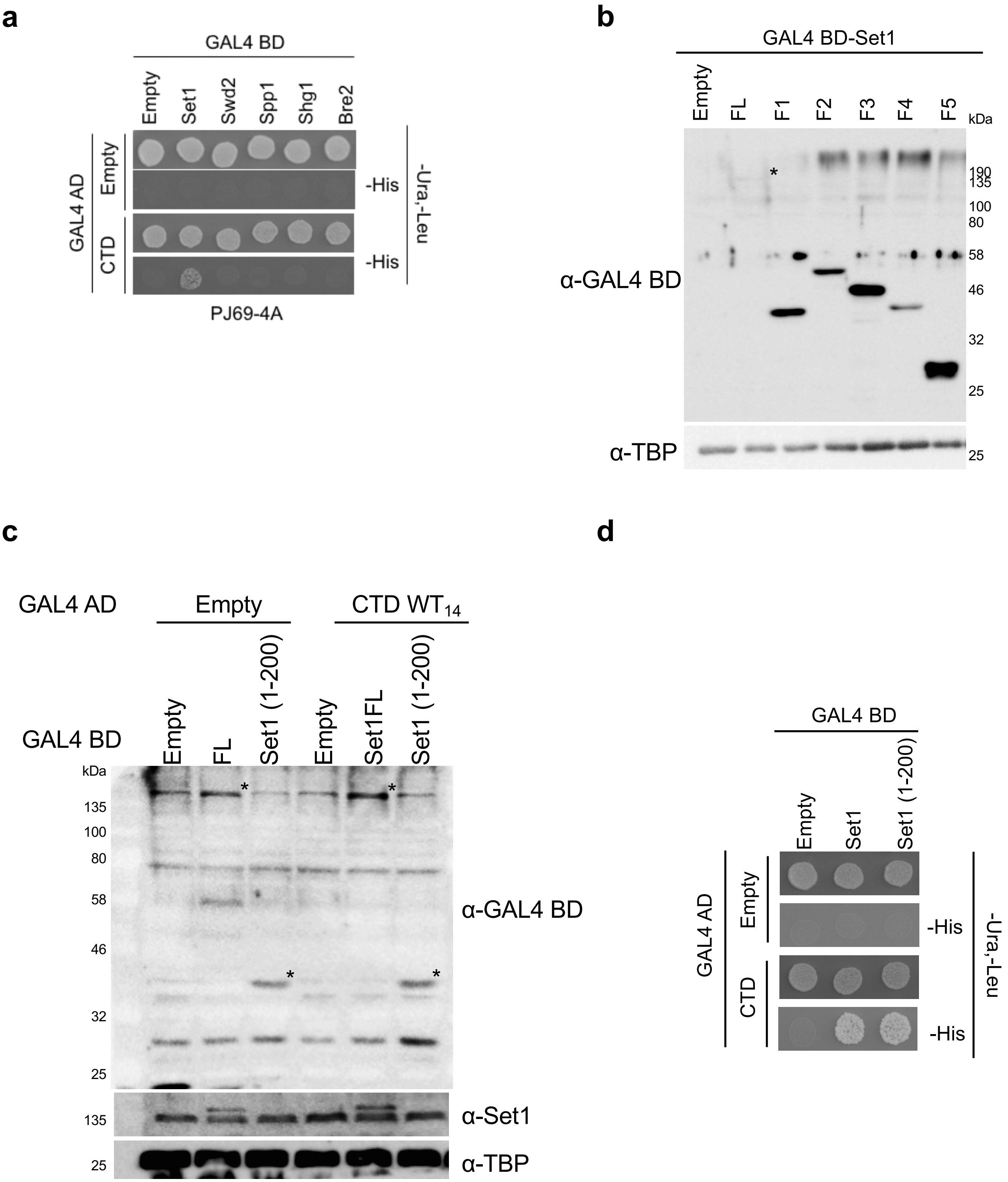

Supplementary Fig. 1

a Plasmids expressing GAL4 BD fused to the indicated COMPASS subunits were transformed into Y2H strain PJ69-4A expressing GAL4 AD alone (Empty) or fused to the Rpb1 CTD (CTD). Cells were grown for 2 days on SC media plate lacking indicated amino acids. Growth in the absence of histidine (-His) indicates reporter gene activation and Y2H interaction.

b Proteins levels of the Set1 fusion proteins were assayed by immunoblotting using anti-GAL4 BD. TBP was used as an internal loading control.

c Protein expression levels of Set1 and Set1(1-200) GAL4 BD fusions were tested by immunoblotting using indicated antibodies. Asterisks show positions of expressed proteins. Note that a cross-reacting band appears just below Set1 in all samples.

d GAL4 BD fused to full length Set1 (Set1) or the N-terminal 200 amino acids (Set1(1-200)) were co-expressed with GAL4 AD-CTD in the PJ69-4A Y2H strain. Empty plasmids harboring GAL4 AD or GAL4 BD (Empty) were used as negative controls. Cells were grown for 2 days on SC media plate lacking the indicated amino acids.

Source data are provided as a Source Data file.

Supplementary Fig. 2

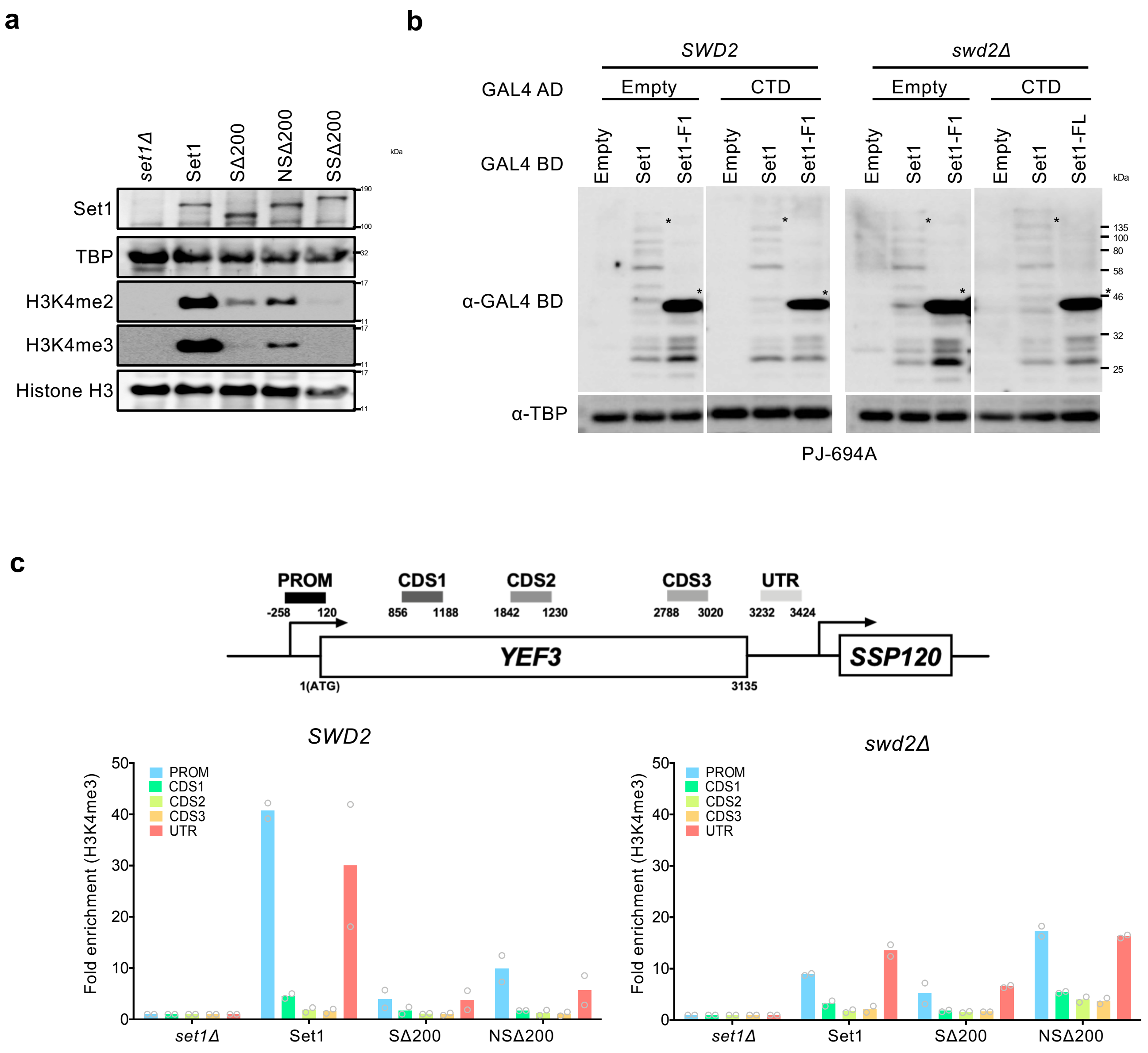

Supplementary Fig. 2

a Immunoblotting was used to detect expression of full-length Set1 (Set1), Set1Δ200 (SΔ200), Nrd1CID-Set1Δ200 (NSΔ200), Swd2-Set1Δ200 (SSΔ200) in a yeast strain with *SET1* deleted (*set1Δ*). Histone H3 K4 di- (H3K4me2) and tri-methylation (H3K4me3) were also tested. TBP and histone H3 were used as loading controls.

b Immunoblots showing expression of full-length Set (Set1) and Set1 N-terminal region (Set1-F1) GAL4 BD fusions expressed in Y2H strain PJ69-4A, either containing (SWD2) or lacking (*swd2Δ*) Swd2. TBP was used as a loading control. Asterisks mark the expected bands.

c ChIP-qPCR analyses of H3K4me3 in *set1Δ* (left panels) or *set1Δswd2Δ* (right panels) strains expressing full-length Set1 (Set1), Set1Δ200 (SΔ200), or Nrd1CID-Set1Δ200 (NSΔ200). Data were normalized to values from cells expressing empty plasmid (*set1Δ)*. Mean values derived from biological replicates (n=2) are represented on the plot.

Source data are provided as a Source Data file.

Supplementary Fig. 3

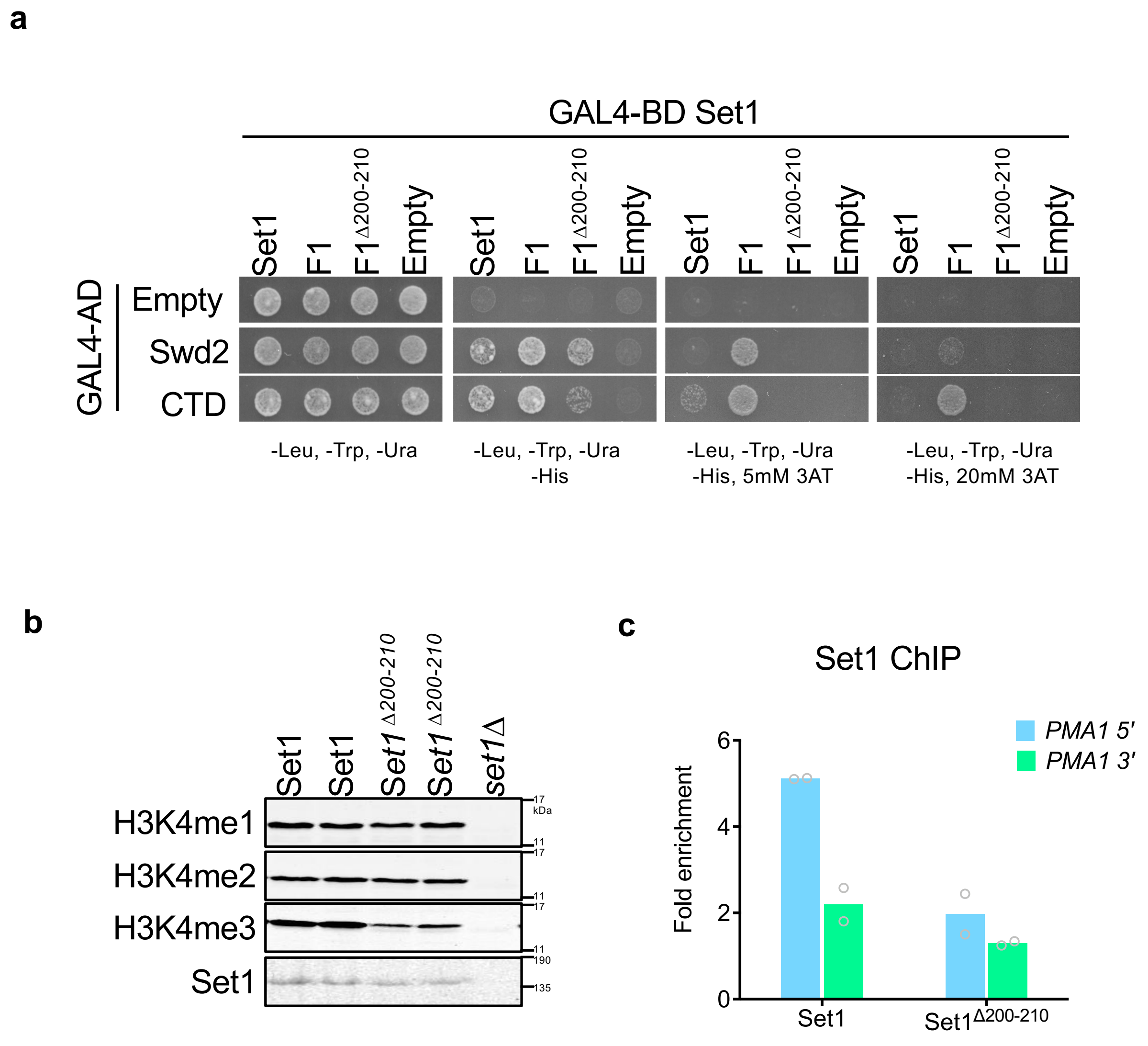

Supplementary Fig. 3

a Constructs expressing GAL4-BD fused to full length Set1 (Set1), Set1 amino acids 1-236 (F1), or the F1 region lacking 11 amino acids ($F1^{\Delta 200\text{-}210}$) were expressed in Y2H strain CG1945 harboring GAL4-AD fused to either Swd2 (Swd2) or the Rpb1 CTD (CTD). Cells were grown for 3 days at 30°C on SC media lacking the indicated amino acids, plus the indicated concentrations of 3-aminotriazol (3AT) to increase stringency of the *HIS3* Gal4 reporter. Empty plasmids were used as a negative control. All spots in each column came from the same plate.

b Full length Set1 (Set1) or the internal deletion $Set1^{\Delta 200\text{-}210}$ (two isolates each) were expressed in a *set1Δ* strain. Levels of Set1 and H3K4 methylations from whole cell extracts were analyzed by immunoblotting with the indicated antibodies.

c Occupancies of Set1 and $Set1^{\Delta 200\text{-}210}$ on the *PMA1* gene were tested using ChIP-qPCR. Data (n=2 biological repeats) were normalized to a non-transcribed sub-telomeric region (TelXV-L).

Source data are provided as a Source Data file.

Supplementary Fig. 4

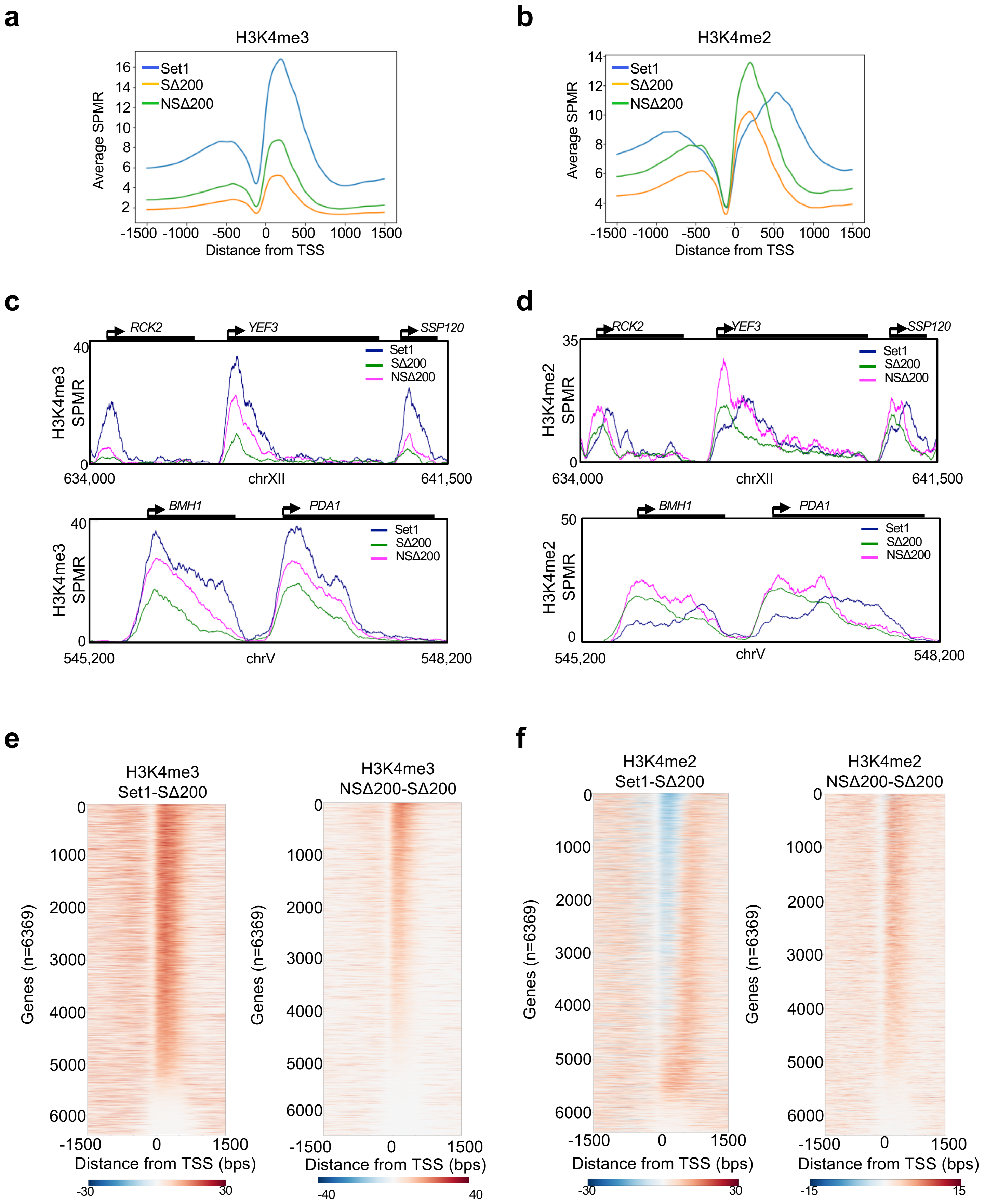

Supplementary Fig. 4

a, b Meta-gene anchor plots of H3K4me3 **(a)** and H3K4me2 **(b)** from wild-type Set1 (blue, Set1), Set1Δ200 (orange, SΔ200), and Nrd1CID-Set1Δ200 (green, NSΔ200) transformed into *set1Δ* cells. ChIP-seq SPMR values for nucleotide positions between -1500 and +1500 were averaged and plotted.

c, d Representative ChIP-Seq browser tracks. SPMR values for H3K4me3 **(c)** and H3K4me2 **(d)** were plotted from chromosome XII region 628,447 to 652,484 (upper panels) and chromosome V region 545,200 to 548,200 (lower panels) using Mochiview2 v1.45 (see Methods).

e, f Heat maps of H3K4me3 **(e)** and H3K4me2 **(f)** represent the difference matrix from subtraction of SΔ200 from Set1 (left panels) or NSΔ200 (right panels). As shown in the scale below each heatmap, red indicates an increase and blue a decrease relative to SΔ200.

Supplementary Fig. 5

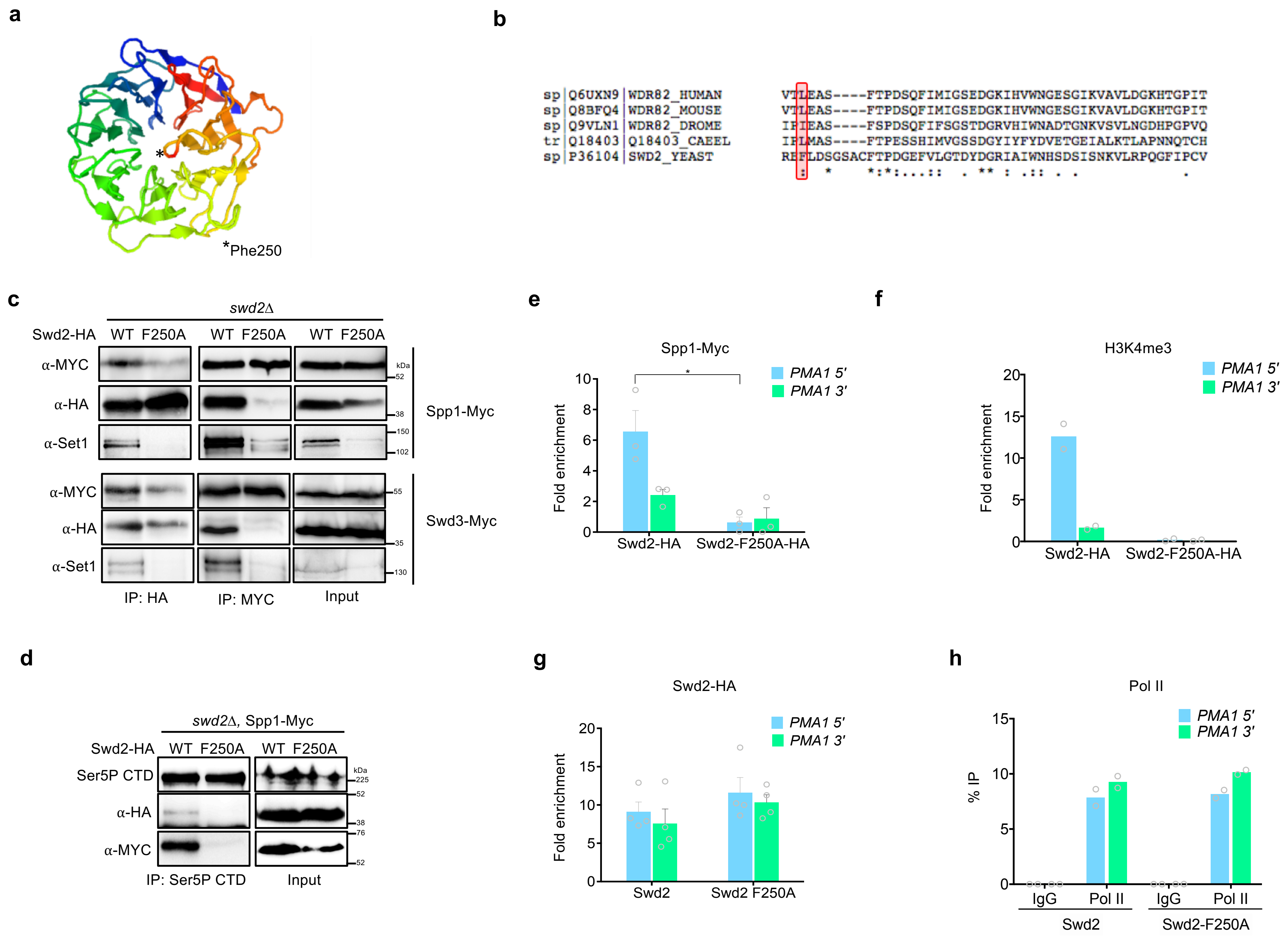

Supplementary Fig. 5

a Model of Swd2 protein structure based on residues 12 to 328 (Swd2: 1-329), created by SWISS-MODEL. The position of the mutated phenylalanine (Phe250) residue replaced by alanine (F250A) is indicated by an asterisk.

b Sequence alignment of Swd2 protein homologs from Clustal 2.1. *WDR82* from Human (Q6UXN9), Mouse (Q8BFQ4), and *Drosophila melanogaster* (Q9VLN1), *Q18403* from *Caenorhabditis elegans* (Q18403) and *SWD2* from *Saccharomyces cerevisiae* (P36104).

c Swd2-HA and Swd2-F250A-HA were expressed in *swd2Δ* strains that also expressed Spp1-MYC (upper panels) or Swd3-MYC (lower panels). Proteins co-immunoprecipitated using anti-MYC (IP:MYC) or anti-HA (IP:HA) were analyzed by immunoblotting with the indicated antibodies.

d Swd2-HA and Swd2-F250A-HA were expressed in *swd2Δ* deletion and Spp1-MYC expressing cells. Immunoprecipitated proteins using monoclonal antibody 3E8 against CTD Ser5P (IP: Ser5P CTD) were analyzed by immunoblotting using indicated antibodies.

e, f ChIP-qPCR analyses of Spp1-MYC **(e)** and H3K4me3 (**f)** enrichment at the *PMA1* gene. Chromatin prepared from cells expressing Swd2-HA WT or Swd2-F250A-HA was immunoprecipitated with antibodies against the MYC tag on Spp1 **(e)** or H3K4me3 **(f)**. Values for primers near the 5' and 3' ends were normalized to those from an untagged strain and non-specific IgG-coated beads. Mean values from biological triplicates (e, mean ± s.e.m. * $p = 0.0143$) or duplicates (f) are represented on the plot. Unpaired t test was used for statistical analysis.

g, h ChIP-qPCR analyses of **(g)** Swd2-HA and **(h)** Rpb1 enrichment at *PMA1* were performed in strains expressing Swd2-HA WT or Swd2-F250A-HA. Chromatin was immunoprecipitated with antibodies against **(g)** the HA tag or **(h)** the Rpb1 CTD (4H8). Untagged strains and IgG-coated beads were used as negative controls for normalization. Chromatin associated DNA was analyzed by quantitative PCR. Mean values from four (g, mean ± s.e.m.) or two (h) biological repeats are represented on the plot.

Source data are provided as a Source Data file.

Supplementary Fig. 6

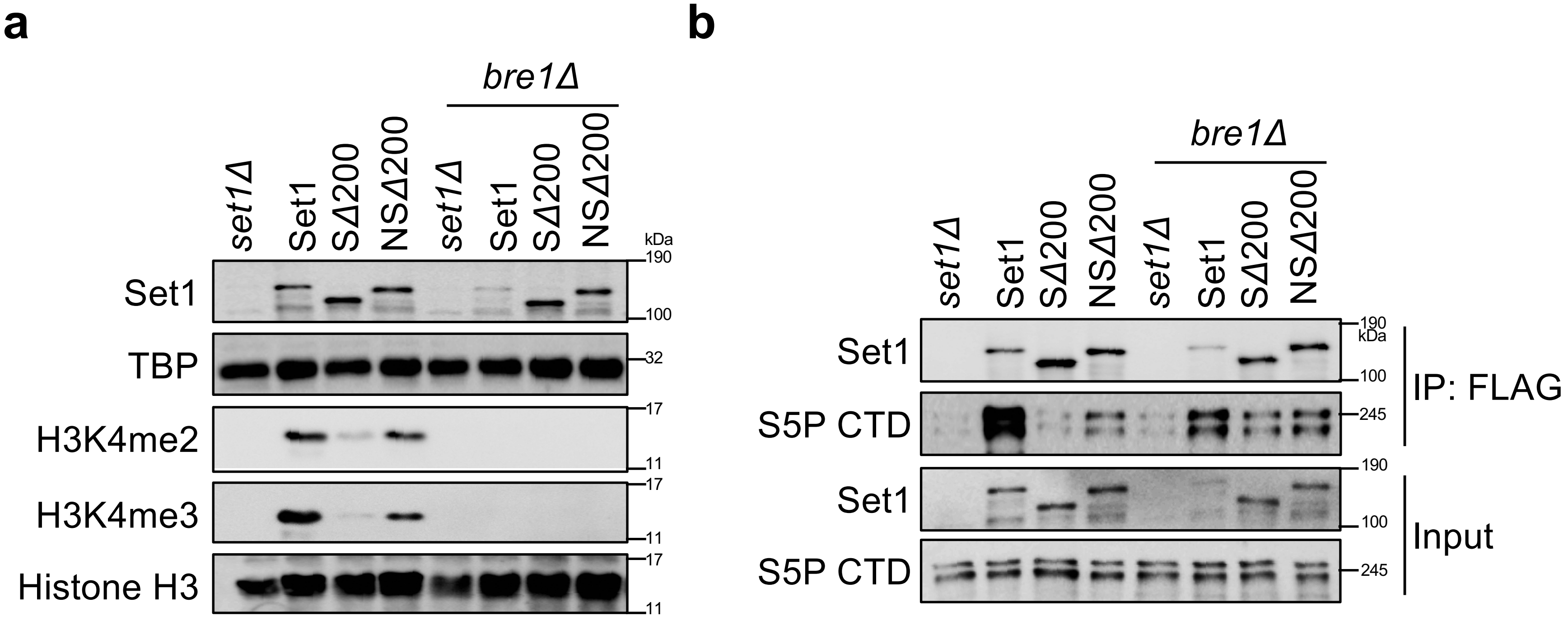

Supplementary Fig. 6

a-b Flag-tagged full length Set1 (Set1), Set1Δ200 (SΔ200), or Nrd1CID-Set1Δ200 (NSΔ200) were transformed into *set1Δ* or *set1Δbre1Δ* cells. Protein and histone methylation levels **(a)** and FLAG-immunoprecipitated proteins **(b,** IP:FLAG**)** were analyzed by immunoblotting. TBP and Histone H3 were used as loading controls.

Source data are provided as a Source Data file.

Supplementary Fig. 7

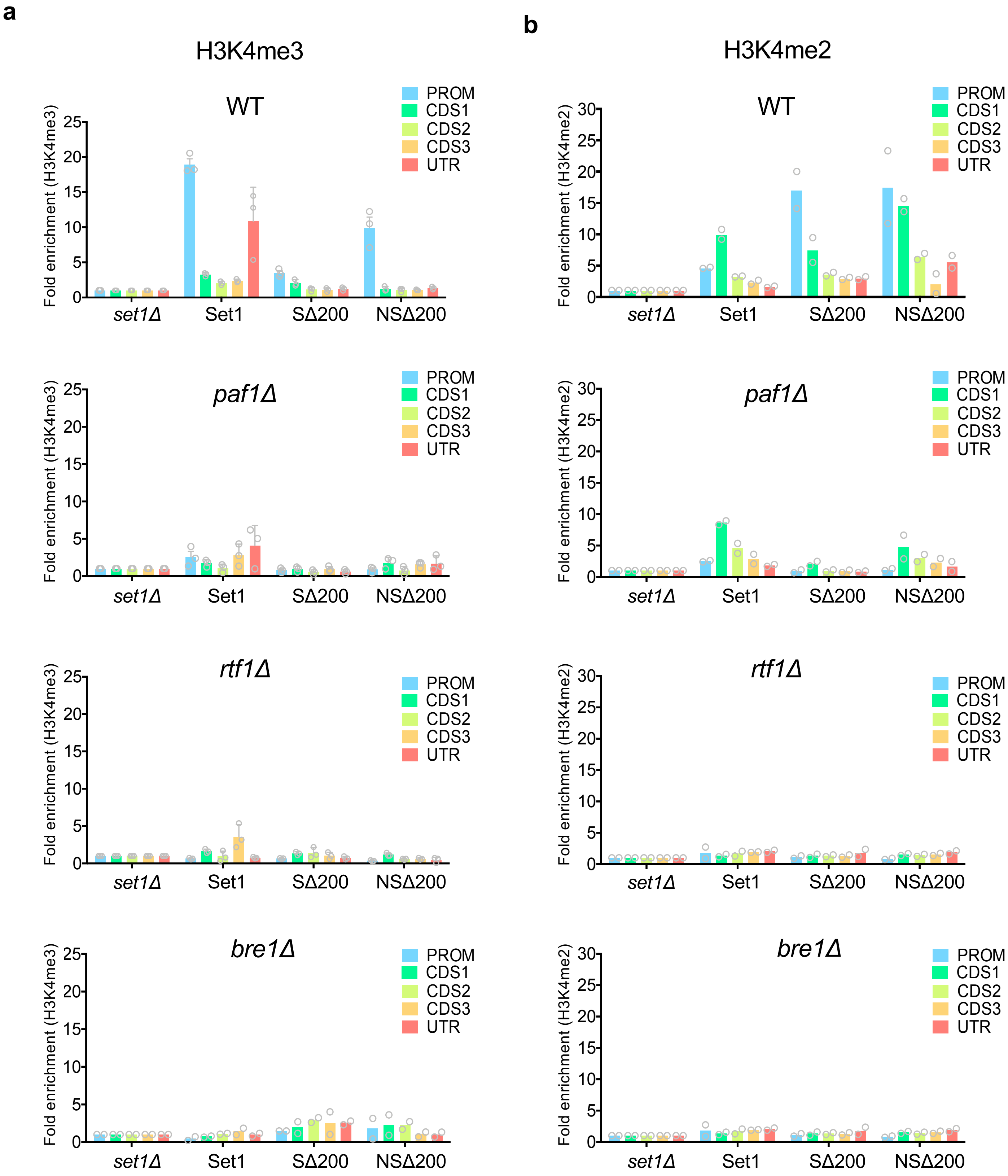

Supplementary Fig. 7

ChIP-qPCR analyses of *set1Δ*, *set1Δpaf1Δ, set1Δrtf1Δ*, and *set1Δbre1Δ* strains expressing full-length Set1 (Set1), Set1Δ200 (SΔ200), and Nrd1CID-Set1Δ200 (NSΔ200). Chromatin preparations from cells expressing each protein were immunoprecipitated with antibodies against **(a)** H3K4me3 or **(b)** H3K4me2, respectively. Data were normalized to values from cells expressing empty plasmid (*set1Δ)*. Error bars represent s.e.m. of multiple independent experiments. Mean values from three (a, *set1Δ*, *set1Δpaf1Δ, set1Δrtf1Δ*, mean ± s.e.m.) or two (a, *set1Δbre1Δ; b*) biological repeats are represented on the plot.

Supplementary Table 1. Yeast strains used in this study

Strain	Genotype	Source or Reference
YSB2613	*MATa, ura3Δ0, leu2Δ0, his3Δ1, met15Δ0, set1Δ::KanMX*	(Soares and Buratowski, 2012)
YSB2718	*MATa, ura3Δ0, leu2Δ0, trp1Δ::LEU2/Kan^R, his3Δ1, met15Δ0, set1Δ::KanMX*	This study
YSB3602	*MATa, ura3Δ0, leu2Δ0, trp1Δ::LEU2/Kan^R, his3Δ1, met15Δ0, set1Δ::HIS3MX, swd2Δ::KanMX,[pSEN1(202)]*	This study
YSB2818	*MATa, ura3Δ0, leu2Δ0, his3Δ1, met15Δ0, rad6Δ::KanMX, set1Δ::HIS3MX*	This study
YSB3028	*MATa, ura3Δ0, leu2Δ0, his3Δ1, met15Δ0, bre1Δ::KanMX, set1Δ::HIS3MX*	This study
YSB3030	*MATa, ura3Δ0, leu2Δ0, his3Δ1, met15Δ0, rtf1Δ::KanMX, set1Δ::HIS3MX*	Soares et al. (2014)
YSB3036	*MATα, ura3Δ0, leu2Δ0, his3Δ1, lys2Δ0, paf1Δ::KanMX, set1Δ::HIS3MX*	Soares et al. (2014)
PJ69-4A	*MATa, ura3-52, leu2-3,112, trp1-901, his3Δ200, gal4Δ, gal80Δ, GAL2-ADE2, LYS2::GAL1-HIS3, met2::GAL7-lacZ*	James et al. (1996)
YSB3457	*MATa, ura3-52, leu2-3,112, trp1-901, his3Δ200, gal4Δ, gal80Δ, GAL2-ADE2, LYS2::GAL1-HIS3, met2::GAL7-lacZ, swd2Δ::KanMX [pSen1(202)]*	This study
CG1945	*MATa, ura3-52, his3-200, lys2-801, ade2-101, trp1-901, leu2-3,112, gal4-542, gal80-538, cyhr2, LYS2::GAL1UAS-GAL1TATA-HIS3, URA3::GAL417mers(X3)-CYC1TATA-lacZ*	Pause et al. (1999)
YMD65	*MATα, leu2-3,112 trp1-1 can1-100 ura3-1 ade2-1 his3-11,15 swd2Δ::KanMX [pSwd2-HA-LEU2]*	This study
YMD68	*MATα, leu2-3,112 trp1-1 can1-100 ura3-1 ade2-1 his3-11,15 swd2Δ::KanMX [pRS415-Swd2-F250A-HA-LEU2]*	This study
YMD223	*MATα, leu2-3,112 trp1-1 can1-100 ura3-1 ade2-1 his3-11,15 swd2Δ::KanMX, Spp1-Myc-HPH [pSwd2-HA-LEU2]*	This study
YMD215	*MATα, leu2-3,112 trp1-1 can1-100 ura3-1 ade2-1 his3-11,15 swd2Δ::KanMX, Spp1-Myc-HPH[pRS415-Swd2-F250A-HA-LEU2]*	This study
KDL701	*MATα, leu2-3,112 trp1-1 can1-100 ura3-1 ade2-1 his3-11,15 set1Δ::KanMX*	Delamarre et al. submitted
YMD77	*MATα, leu2-3,112 trp1-1 can1-100 ura3-1 ade2-1 his3-11,15 set1Δ::KanMX, [pSet1-LEU2]*	This study
YMD73	*MATα, leu2-3,112 trp1-1 can1-100 ura3-1 ade2-1 his3-11,15 set1Δ::KanMX [pSet1$^{\Delta 200\text{-}210}$-LEU2]*	This study
YSB3603	*MATa, ura3-52, leu2-3,112, trp1-901, his3Δ200, gal4Δ, gal80Δ, GAL2-ADE2, LYS2::GAL1-HIS3, met2::GAL7-lacZ, swd2Δ::KanMX, [pRS416-Swd2-HA]*	This study
YSB3604	*MATa, ura3-52, leu2-3,112, trp1-901, his3Δ200, gal4Δ, gal80Δ, GAL2-ADE2, LYS2::GAL1-HIS3, met2::GAL7-lacZ swd2Δ::KanMX, [pRS416-Swd2 F250A-HA]*	This study

Supplementary Table 2. Plasmids used in immunoblotting and co-IP

Plasmid	Genotype	Source or Reference
pRS416-SET1 (1-1080)	ADH1 promoter driving SET1 (1-1080) with N-terminal FLAG tag, URA3, CEN/ARS, f1+ ori, AmpR	Fingerman et al. (2005)
pRS416-SET1Δ100	ADH1 promoter driving SET1 Δ100 with N-terminal FLAG tag, URA3, CEN/ARS, f1+ ori, AmpR	Soares et al. (2014)
pRS416-SET1Δ200	ADH1 promoter driving SET1 Δ200 with N-terminal FLAG tag, URA3, CEN/ARS, f1+ ori, AmpR	Soares et al. (2014)
pRS416-SET1Δ700	ADH1 promoter driving SET1 Δ700 with N-terminal FLAG tag, URA3, CEN/ARS, f1+ ori, AmpR	Soares et al. (2014)
pRS416-Nrd1CID-SET1Δ100	ADH1 promoter driving Nrd1CID-SET1 Δ100 with N-terminal FLAG tag, URA3, CEN/ARS, f1+ ori, AmpR	This study
pRS416-Nrd1CID-SET1Δ200	ADH1 promoter driving Nrd1CID-SET1 Δ200 with N-terminal FLAG tag, URA3, CEN/ARS, f1+ ori, AmpR	This study
pRS416-Nrd1CID(D70R)-SET1 Δ200	ADH1 promoter driving Nrd1CID (D70R)-SET1 Δ200 with N-terminal FLAG tag, URA3, CEN/ARS, f1+ ori, AmpR	This study
pRS416-Nrd1CID(I130R)-SET1 Δ200	ADH1 promoter driving Nrd1CID(I130R)-SET1 Δ200 with N-terminal FLAG tag, URA3, CEN/ARS, f1+ ori, AmpR	This study
pRS414-SET1 (1-1080)	ADH1 promoter driving SET1 (1-1080) with N-terminal FLAG tag, TRP1, CEN/ARS, f1+ ori, AmpR	Fingerman et al. (2005)
pRS414-SET1Δ200	ADH1 promoter driving SET1 Δ200 with N-terminal FLAG tag, TRP1, CEN/ARS, f1+ ori, AmpR	This study
pRS414-Nrd1CID-SET1Δ200	ADH1 promoter driving Nrd1CID-SET1 Δ200 with N-terminal FLAG tag, TRP1, CEN/ARS, f1+ ori, AmpR	This study
pRS415-Set1	ADH1 promoter driving SET1, LEU2, CEN/ARS, f1+ ori, AmpR	This study
pRS415-Set1$^{\Delta 200\text{-}210}$	ADH1 promoter driving SET1Δ200-210, LEU2, CEN/ARS, f1+ ori, AmpR	This study
pRS415-Swd2-HA	ADH1 promoter driving SWD2 with C-terminal HA tag, LEU2, CEN/ARS, f1+ ori, AmpR	This study
pRS415-Swd2-F250A-HA	ADH1 promoter driving SWD2-F250A with C-terminal HA tag, LEU2, CEN/ARS, f1+ ori, AmpR	This study

Supplementary Table 3. Plasmids used in yeast two hybrid assay

Plasmid	Genotype	Source or Reference
pP6	ADH1 promoter driving GAL4 activation domain, LEU2, 2µ ori, AmpR	Hybrigenics
pP6-CTD_{14}	ADH1 promoter driving GAL4 activation domain-RPO21 CTD (14 repeats), LEU2, 2µ ori, AmpR	This study
pP6-Swd2	ADH1 promoter driving GAL4 activation domain-Swd2, LEU2, 2µ ori, AmpR	This study
pB66	ADH1 promoter driving GAL4 DNA binding domain, TRP1, 2µ ori, KanR	Hybrigenics
pB66-Set1 FL	ADH1 promoter driving GAL4 DNA binding domain-SET1 (Full length), TRP1, 2µ ori, KanR	This study
pB66-Set1 F1	ADH1 promoter driving GAL4 DNA binding domain-SET1 F1 (aa 1-236), TRP1, 2µ ori, KanR	This study
pB66-Set1 F2	ADH1 promoter driving GAL4 DNA binding domain-SET1 F2 (aa 240-586), TRP1, 2µ ori, KanR	This study
pB66-Set1 F3	ADH1 promoter driving GAL4 DNA binding domain-SET1 F3 (aa 590-769), TRP1, 2µ ori, KanR	This study
pB66-Set1 F4	ADH1 promoter driving GAL4 DNA binding domain-SET1 F4 (aa 770-945), TRP1, 2µ ori, KanR	This study
pB66-Set1 F5	ADH1 promoter driving GAL4 DNA binding domain-SET1 F5 (aa 946-1080), TRP1, 2µ ori, KanR	This study
pB66- F1 $Set1^{\Delta 200\text{-}210}$	ADH1 promoter driving GAL4 DNA binding domain-SET1 (1-236 Δ200-210), TRP1, 2µ ori, KanR	This study
pGBDU-C1	ADH1 promoter driving GAL4 DNA binding domain, URA3, 2µ ori, AmpR	James et al. (1996)
pGBDU-Bre2	ADH1 promoter driving GAL4 DNA binding domain-BRE2 (Full length), URA3, 2µ ori, AmpR	This study
pGBDU-Set1	ADH1 promoter driving GAL4 DNA binding domain-SET1 (Full length), URA3, 2 µ ori, AmpR	This study
pGBDU-$Set1_{1\text{-}200}$	ADH1 promoter driving GAL4 DNA binding domain-SET1 (aa 1-200), URA3, 2 µ ori, AmpR	This study
pGBDU-Spp1	ADH1 promoter driving GAL4 DNA binding domain-SPP1 (Full length), URA3, 2 µ ori, AmpR	This study
pGBDU-Shg1	ADH1 promoter driving GAL4 DNA binding domain-SHG1 (Full length), URA3, 2 µ ori, AmpR	This study
pGBDU-Swd2	ADH1 promoter driving GAL4 DNA binding domain-SWD2 (Full length), URA3, 2 µ ori, AmpR	This study
pRS416-Swd2-HA	ADH1 promoter driving SWD2 with C-terminal HA tag, URA3, CEN/ARS, f1+ ori, AmpR	This study
pRS416-Swd2-F250A-HA	ADH1 promoter driving SWD2-F250A with C-terminal HA tag, URA3, CEN/ARS, f1+ ori, AmpR	This study

Supplementary Table 4. Oligonucleotides used in this study

Purpose	Gene		Sequence (5' -> 3')
Reverse PCR	SET1	Forward 1 (Set1Δ100)	ATGTCTCAATCACGGTATTCAAATAGC
		Forward 2 (Set1Δ200)	GAGATAAAAGCATCCGACCCACG
		Reverse	CGCGTGTGCTCTTCTATAGTAATTTGA
Isothermal assembly	NRD1	Forward	AAATTACTATAGAAGAGCACACGCGATGCAGC AGGACGACGAT
		Reverse 1 (to Set1Δ100)	TATTTGAATACCGTGATTGAGACATCTTGGGAT CCAGTGATAGTT
		Reverse 2 (to Set1Δ200)	TACGTGGGTCGGATGCTTTTATCTCCTTGGGAT CCAGTGATAGTT
ChIP-qPCR	YEF3-1	Forward	GCACGTGAAAAAGAAACGTTTTTAATG
		Reverse	GATGTTACCATTCAAGAAAGAAGCGAC
	YEF3-2	Forward	TCTTGGGTAAATTGTTGCCAGG
		Reverse	GTGCAAGAAGATAGTCATGTATGGGGTG
	YEF3-3	Forward	GGTTTGAAGTTGAGAAAGTACAAGGG
		Reverse	TCAAAGTAGACTTACCAGCACC
	YEF3-4	Forward	GATTCTTTGGGTGCTTTGTCTAAGGC
		Reverse	CGGCAATCTTGTTACCCATAGC
	YEF3-5	Forward	TTGGTAAAATATAGACGCAACTTCC
		Reverse	GATCCGTCACCTATTTTTATTCTTCC
	PMA1 5'	Forward	TCAGCTCATCAGCCAACTCAAG
		Reverse	CGTCGACACCGTGATTAGATTG
	PMA1 3'	Forward	TACTGTCGTCCGTGTCTGGATCT
		Reverse	CCTTCATTGGCTTACCGTTCA
	TELVI	Forward	GCGTAACAAAGCCATAATGCCTCC
		Reverse	CTCGTTAGGATCACGTTCGAATCC
	TEL XV-L	Forward	AACCCTGTCCAACCTGTCTCC
		Reverse	ATCGTGGTTCGCTGTGGTAT

Supplementary References

1. Soares, L. M & Buratowski, S. Yeast Swd2 is essential because of antagonism between Set1 histone methyltransferase complex and APT (associated with Pta1) termination factor. *J. Biol. Chem.* **287**: 15219–15231 (2012)

2. Soares, L. M et al., Feedback control of Set1 protein levels is important for proper H3K4 methylation patterns. *Cell reports* **6**: 961-72. (2014)

3. James, P., Halladay, J. & Craig, E.A. Genomic Libraries and a host strain designed for highly efficient two-hybrid seletion in yeast. *Genetics* **144**: 1425-1436 (1996)

4. Pause, A., Peterson, B., Schaffar, G., Stearman, R. & Klausner R. D. Studying interactions of four proteins in the yeast two-hybrid system: structural resemblance of the pVHL/elongin BC/hCUL-2 complex with the ubiquitin ligase complex SKP1/cullin/F-box protein. *Proc Natl Acad Sci USA*. **96**: 9533-9538 (1999)

5. Delamarre, A., Barthe, A., de la Roche Saint-André, C., Luciano, P., Forey, R., Padioleau, I., Skrzypczak, M., Ginalski, K., Géli, V., Lengronne A., and Pasero, P. MRX increases chromatin accessibility at stalled replication forks to promote nascent DNA resection and cohesin loading. *Mol Cell* **77**: 395-410 (2020)

6. Fingerman, I. M., Wu, C-L., Wilson, B. D., & Briggs, S.D. Global loss of Set1-mediated H3 Lys4 trimethylation is associated with silencing defects in Saccharomyces cerevisiae. *J. Biol. Chem.* **280**: 28761-28765 (2005)

7. Soares, L. M, et al., Determinants of Histone H3K4 Methylation Patterns. *Mol Cell* **68**: 773-785 (2017)
