# Supplementary material for: The Set1 N-terminal domain and Swd2 interact with RNA polymerase II CTD to recruit COMPASS
Source: Nat Commun. 2020 May 1;11:2181. doi: 10.1038/s41467-020-16082-2 (PMC7195483; doi:10.1038/s41467-020-16082-2)

Source Data for Bae, Dubarry et al.

The Set1 N-terminal domain and Swd2 interact with RNA polymerase II CTD to recruit COMPASS

Fig. 1b

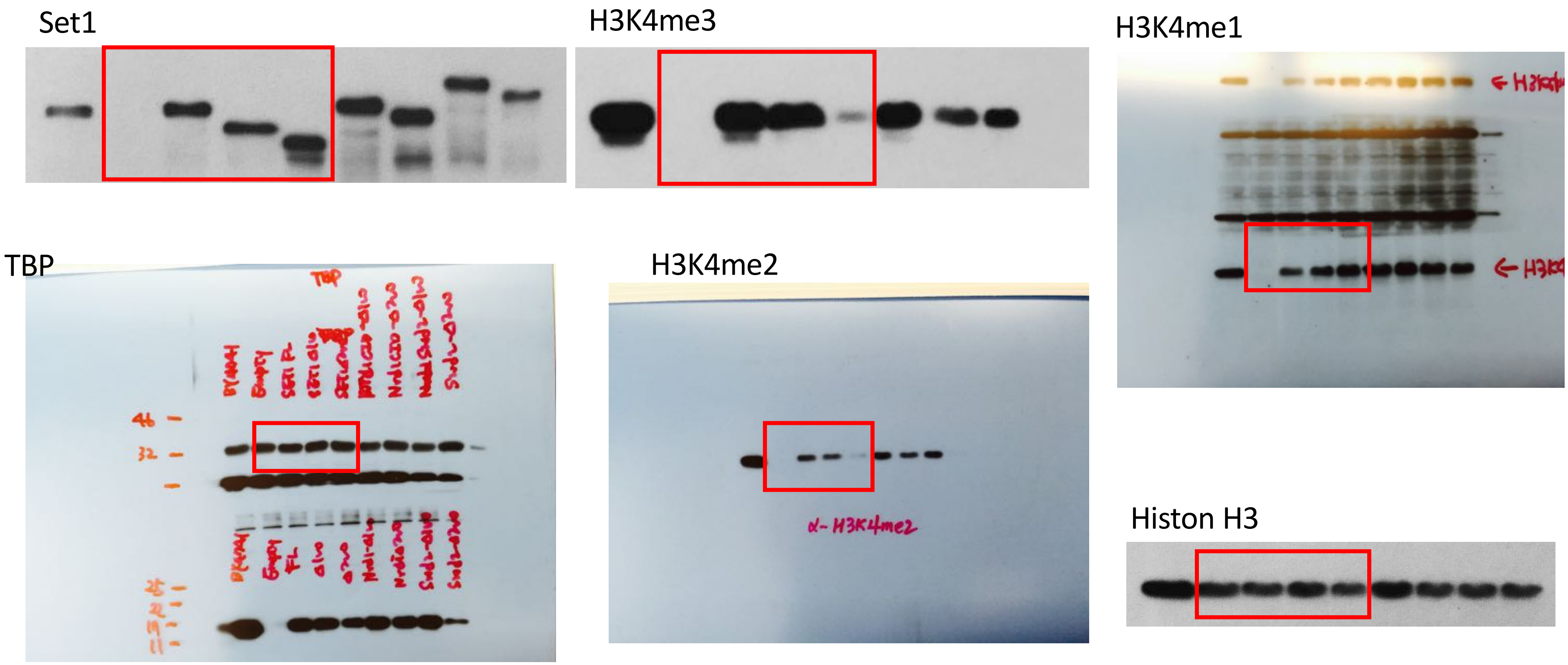

Fig. 1c

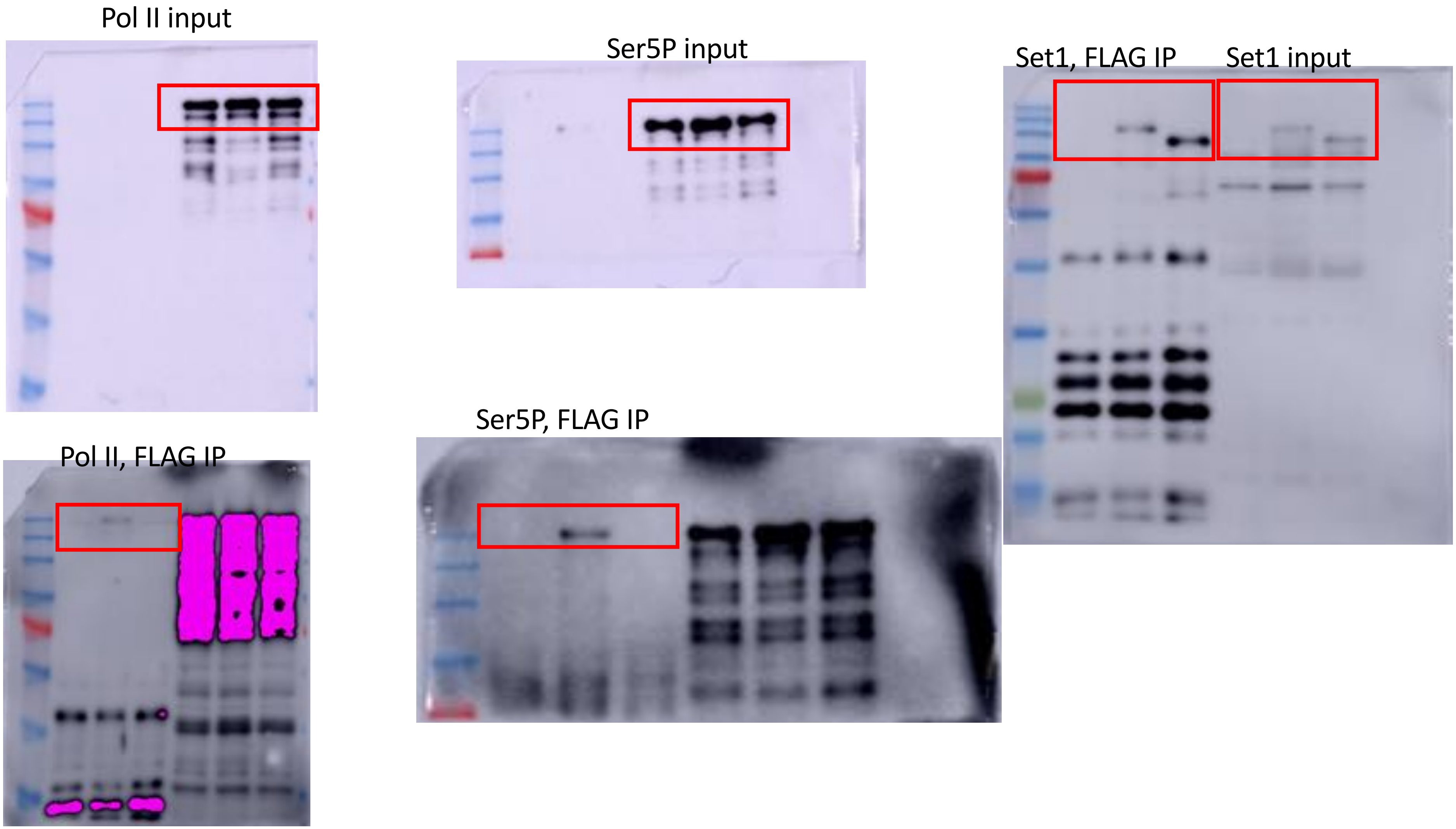
Pol II input
Ser5P input
Set1, FLAG IP
Set1 input
Pol II, FLAG IP
Ser5P, FLAG IP

Fig. 2b

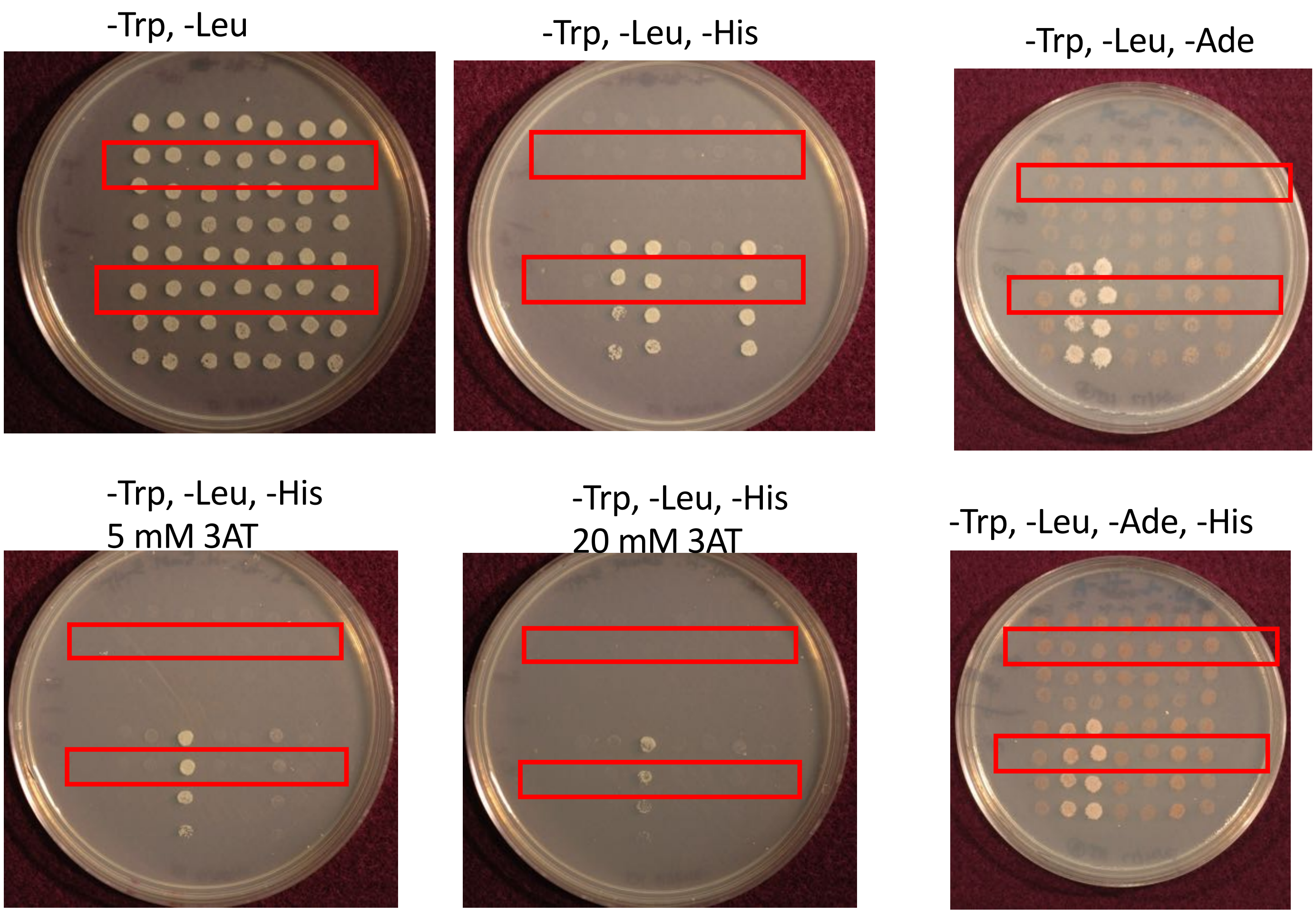
-Trp, -Leu
-Trp, -Leu, -His
-Trp, -Leu, -Ade
-Trp, -Leu, -His
5 mM 3AT
-Trp, -Leu, -His
20 mM 3AT
-Trp, -Leu, -Ade, -His

Fig. 2c

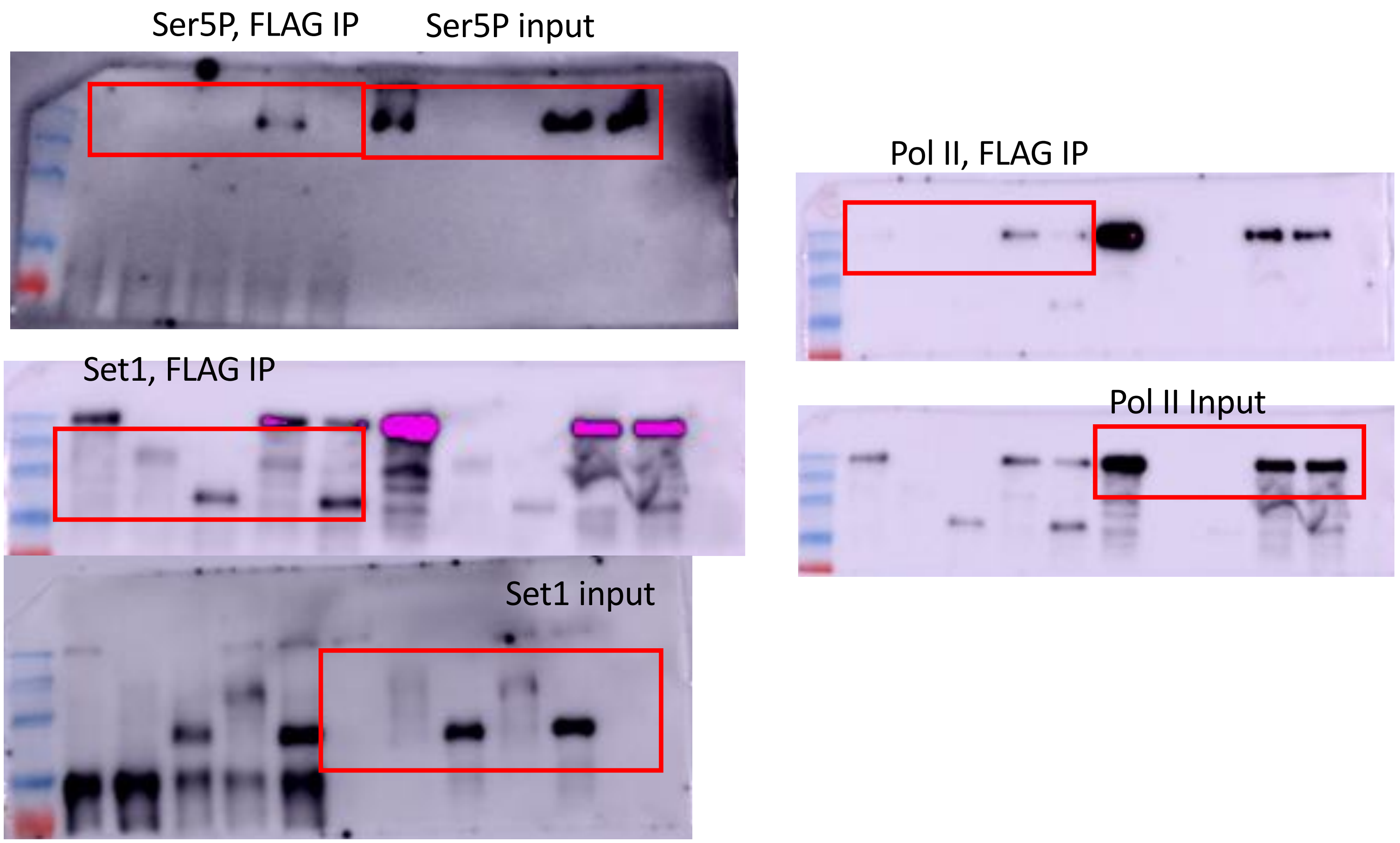
Ser5P, FLAG IP
Ser5P input
Set1, FLAG IP
Set1 input
Pol II, FLAG IP
Pol II Input

Fig. 3

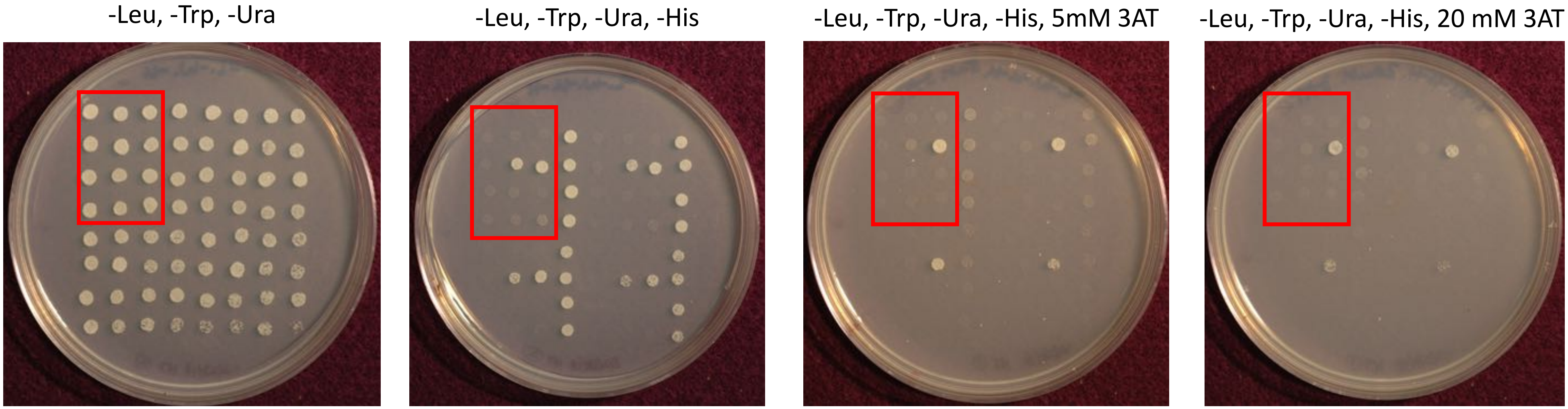
-Leu, -Trp, -Ura
-Leu, -Trp, -Ura, -His
-Leu, -Trp, -Ura, -His, 5mM 3AT
-Leu, -Trp, -Ura, -His, 20 mM 3AT

Fig. 4b

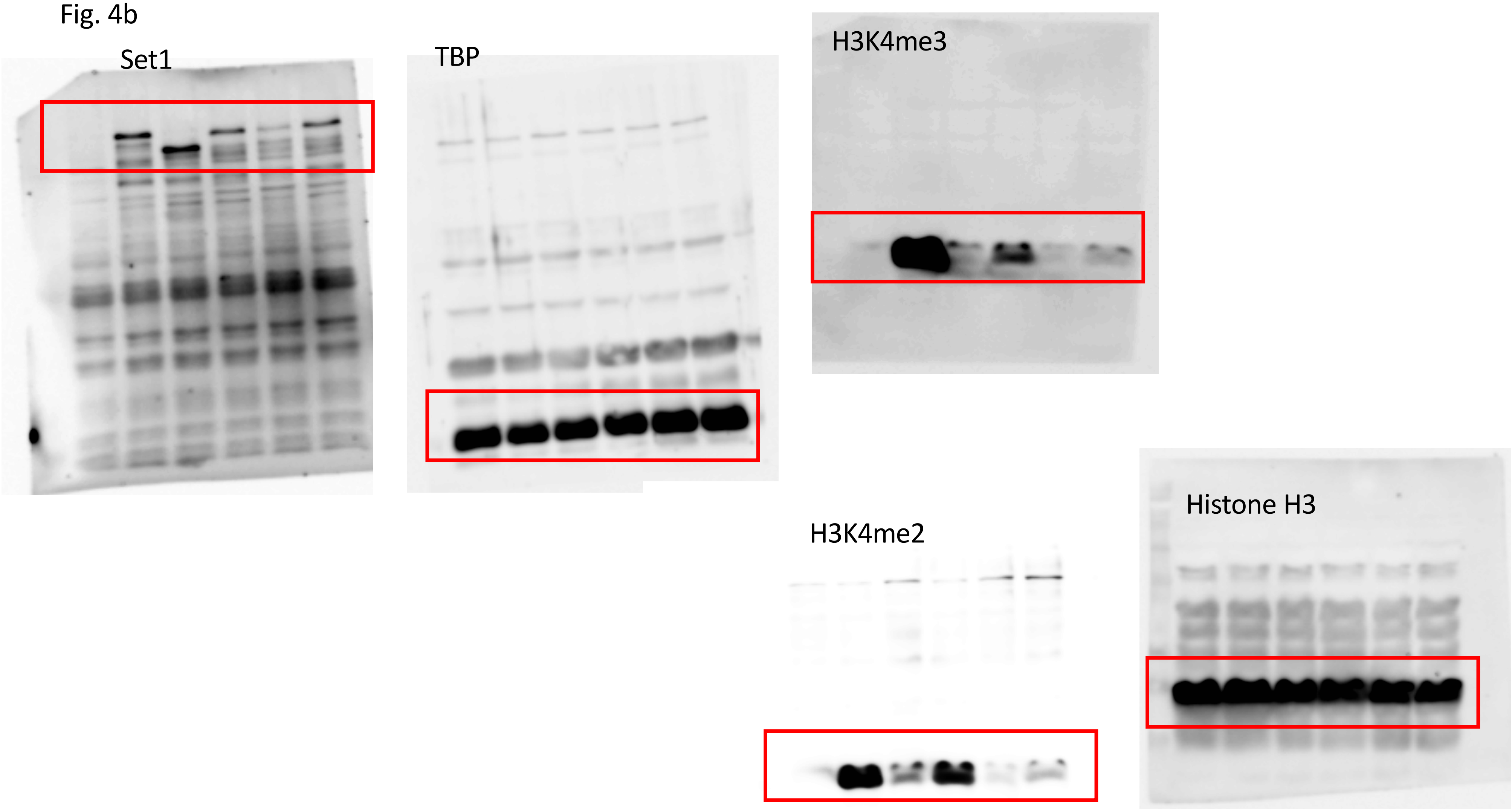

Fig. 4c

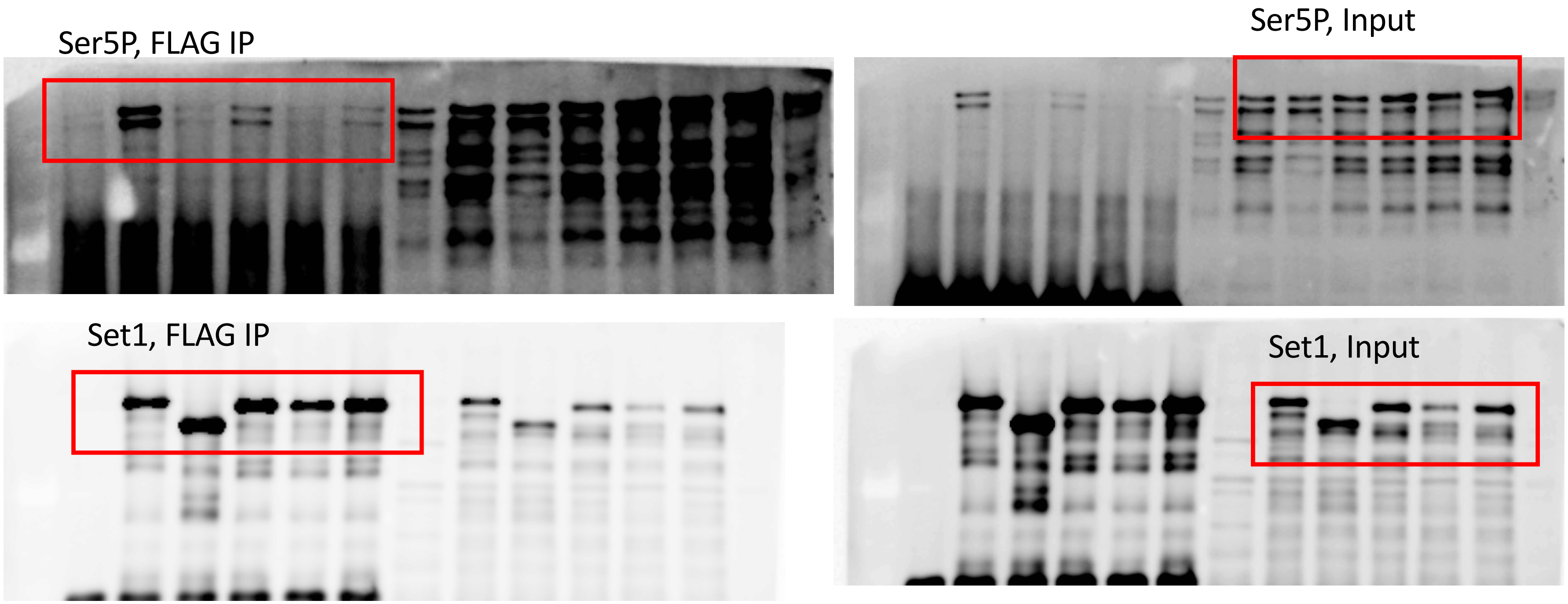

Fig. 4f

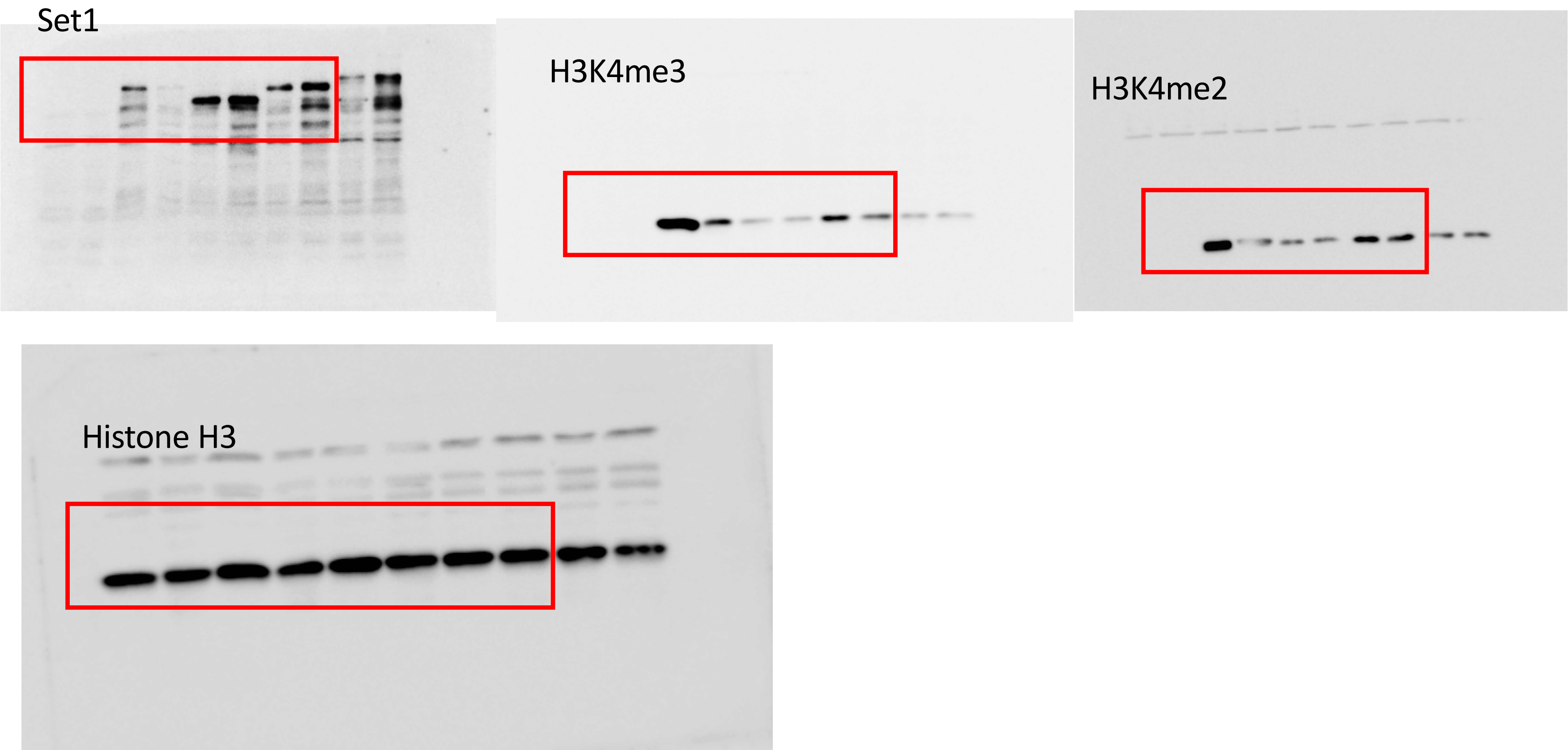

Fig. 4g

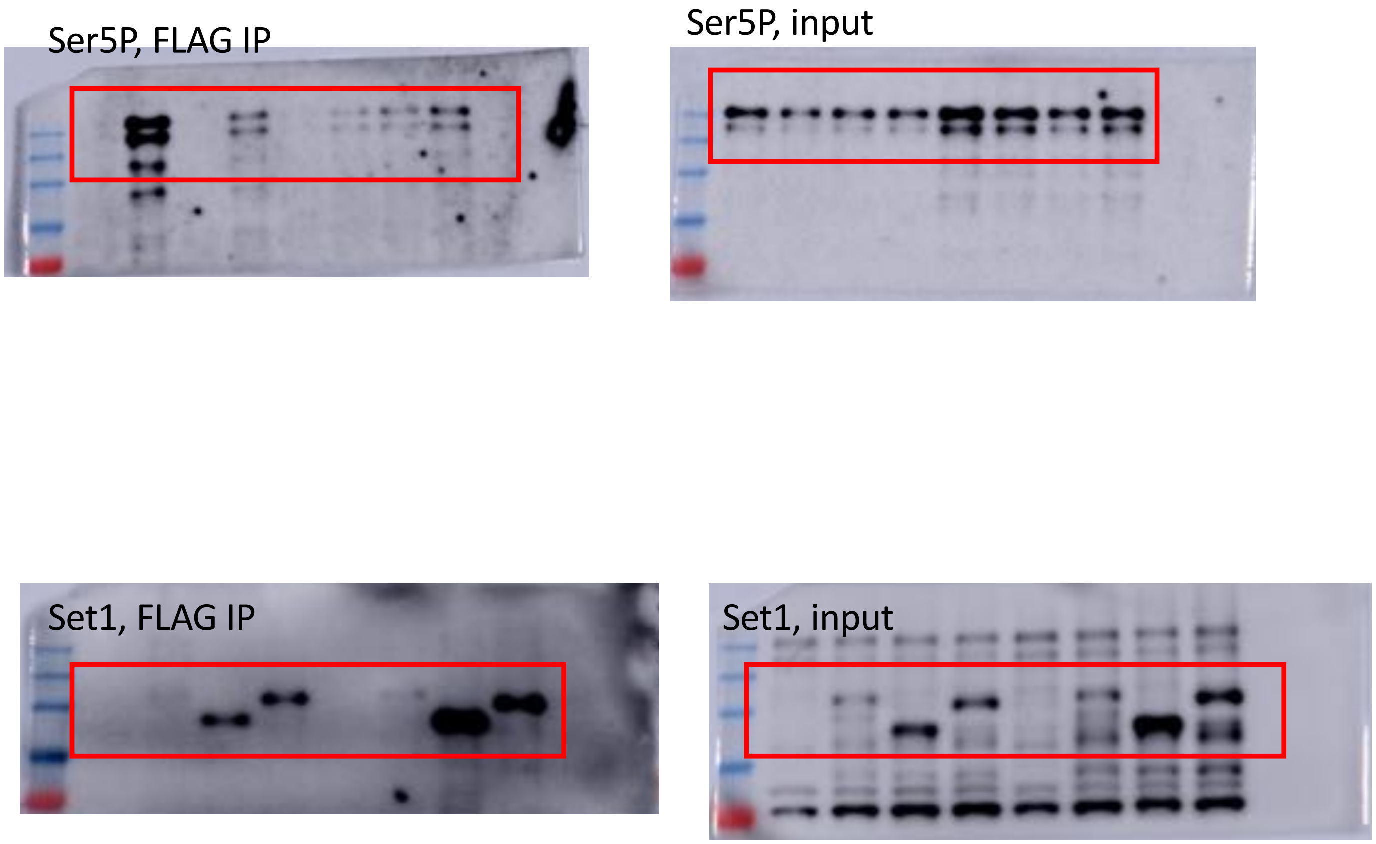

Fig. 5a

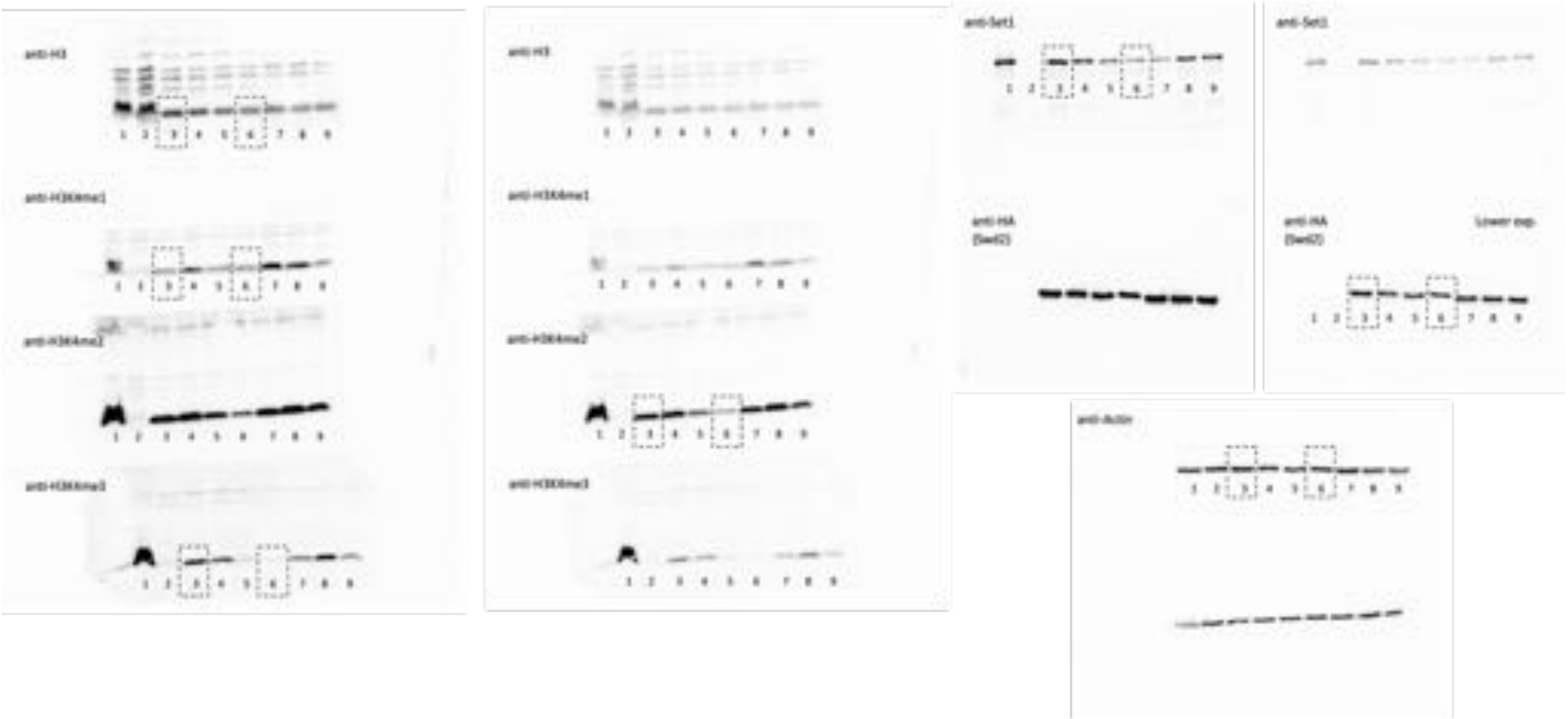

Fig. 5b

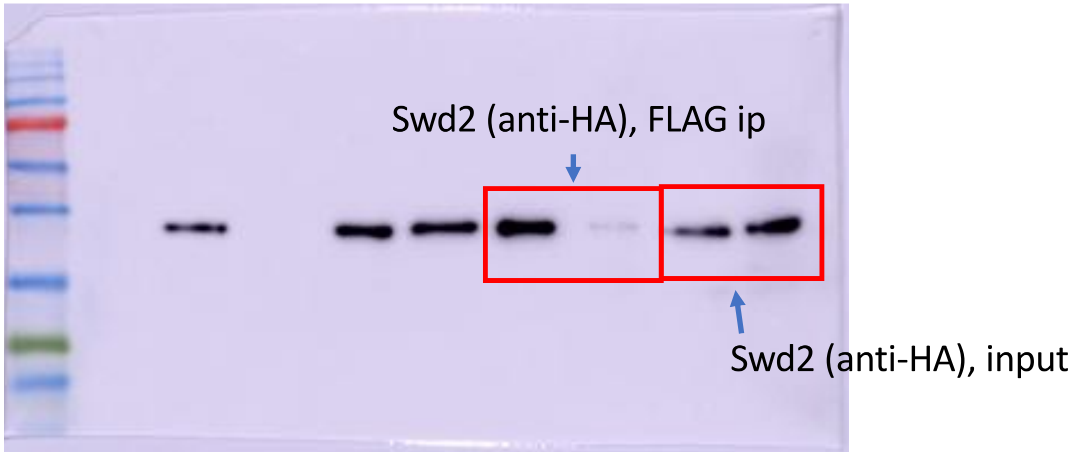
Swd2 (anti-HA), FLAG ip
Swd2 (anti-HA), input

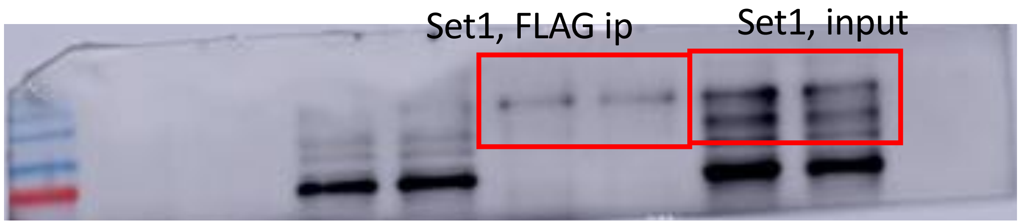
Set1, FLAG ip
Set1, input

Fig. 5c

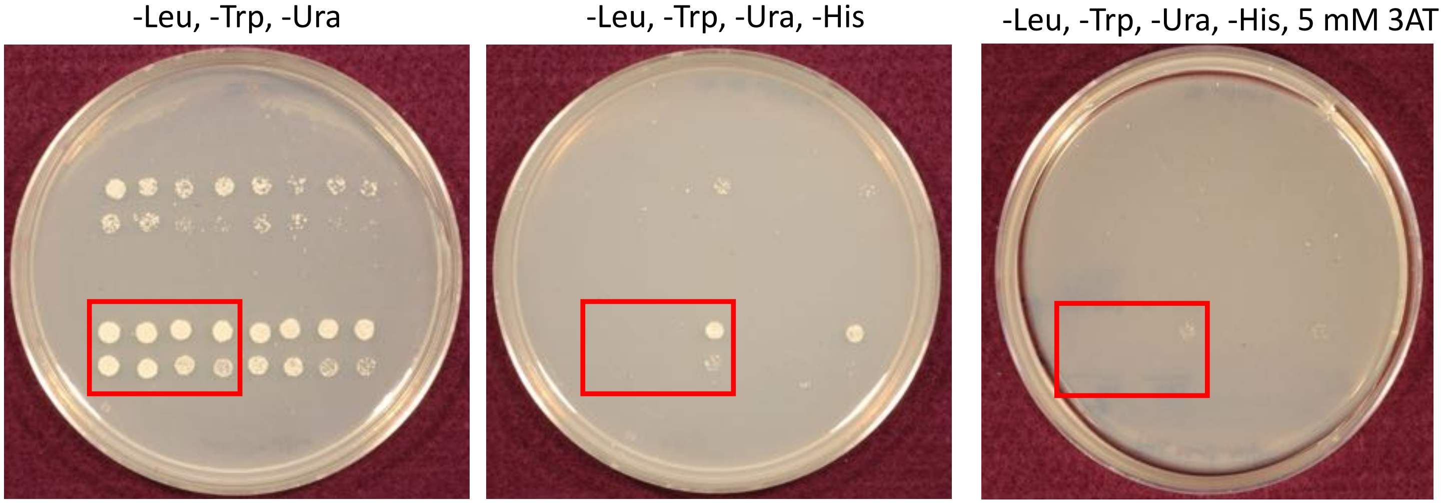

Fig.5d

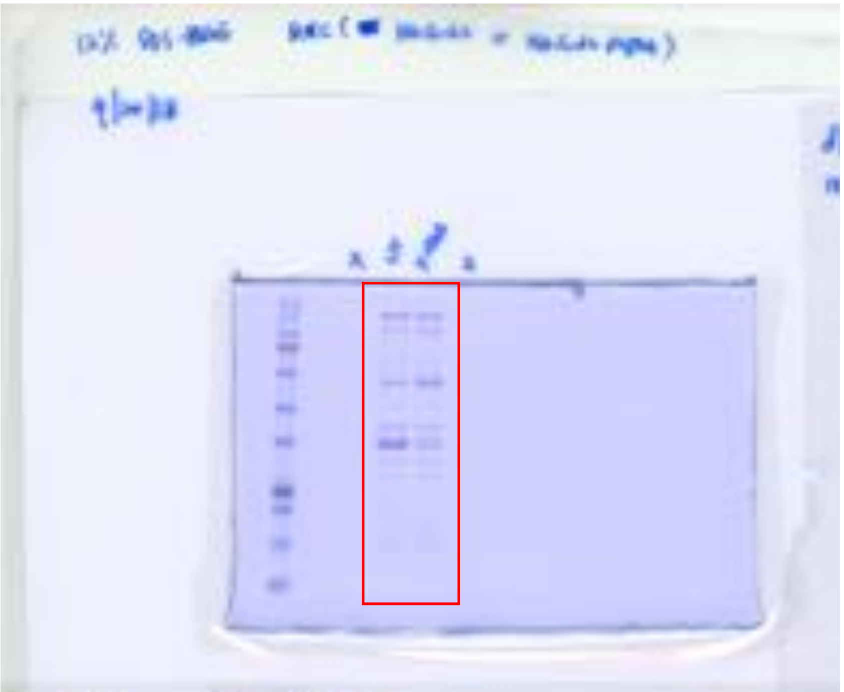

Fig.5e

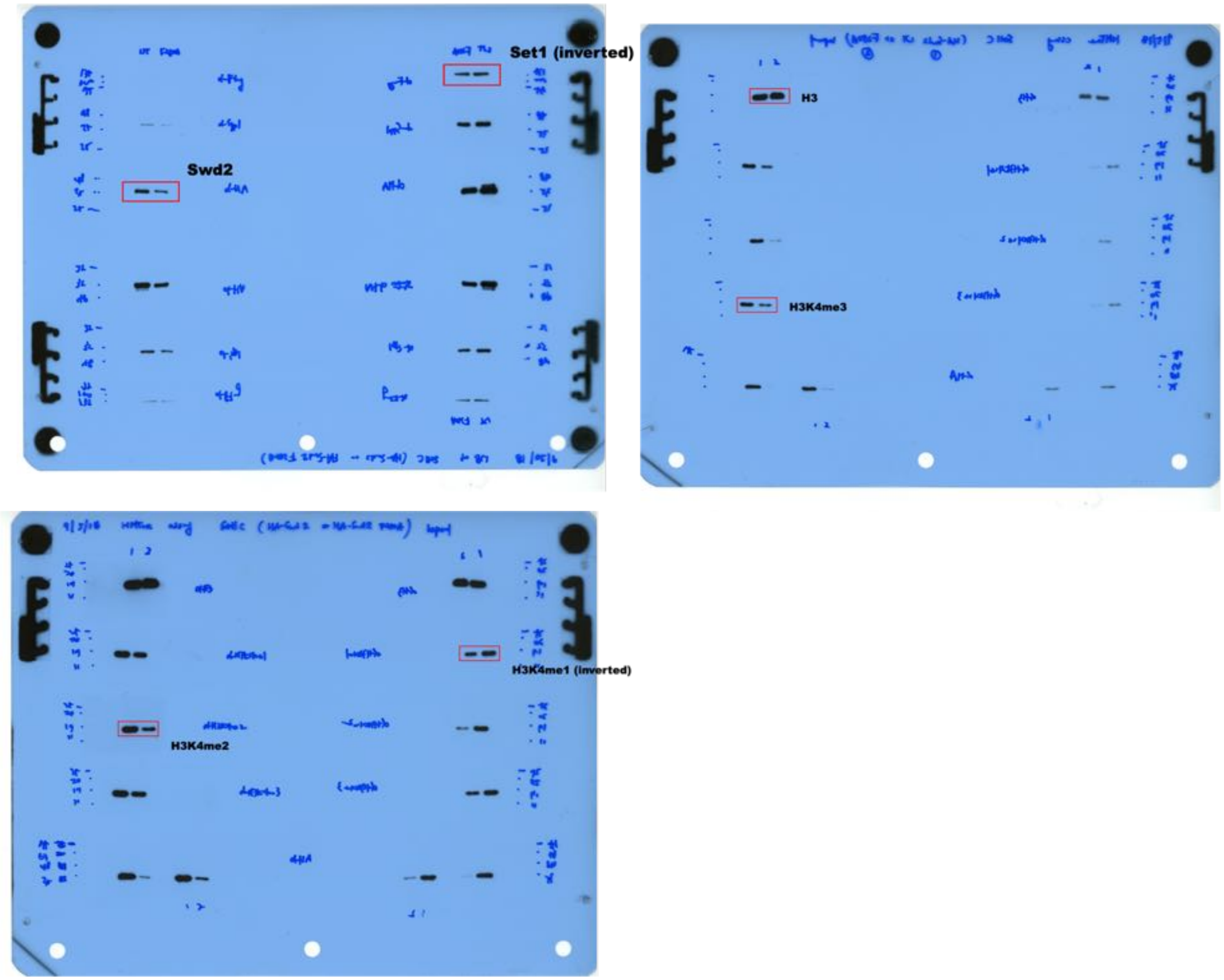

Fig.6a

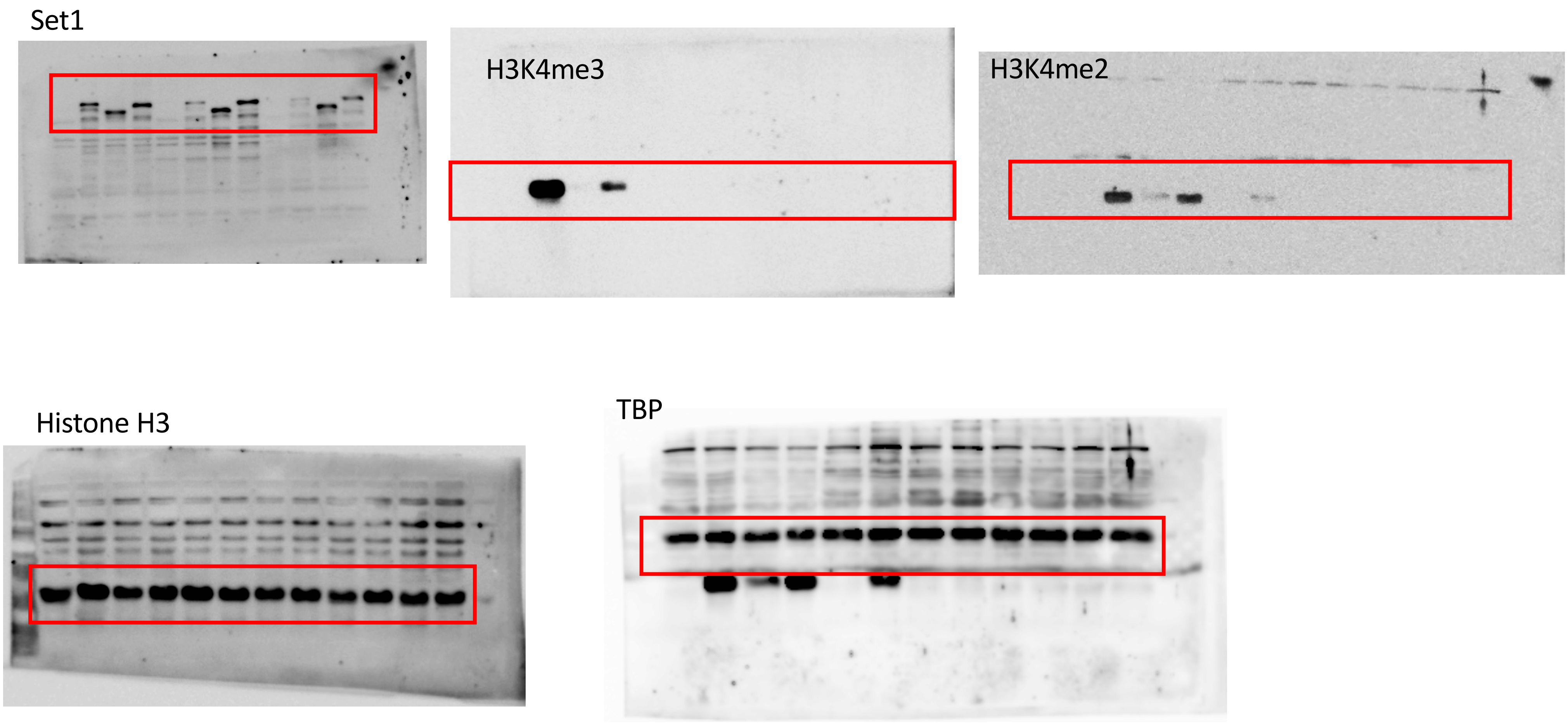

Supplementary Fig. 1a

-Ura, -Leu

-Ura, -Leu, -His

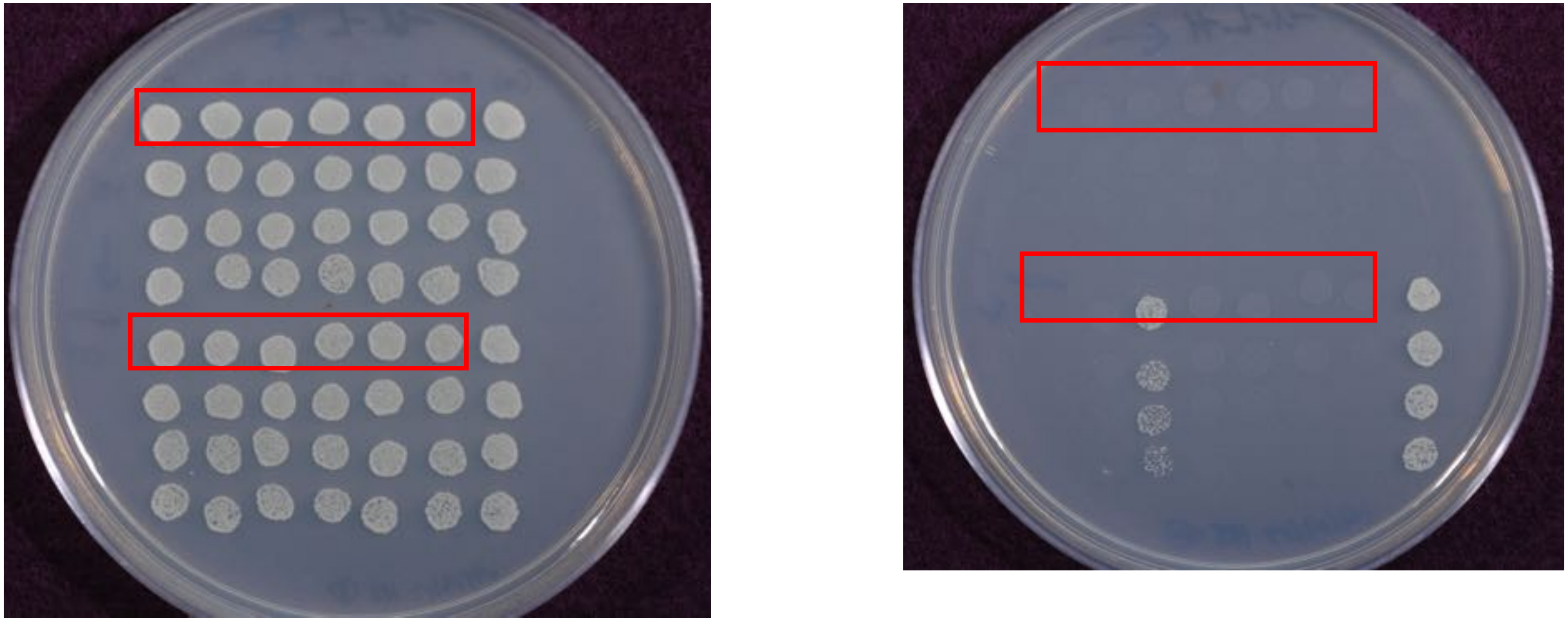

Supplementary Fig. 1b

GAL4BD

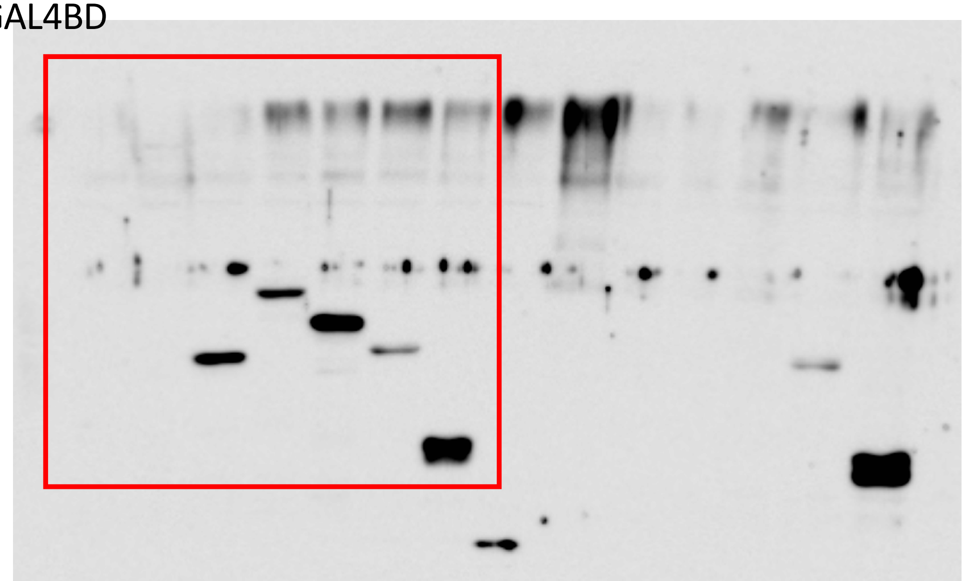

TBP

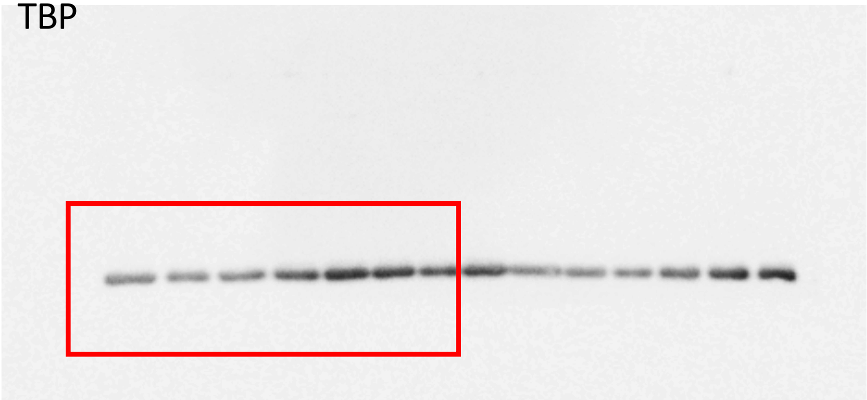

Supplementary Fig. 1c

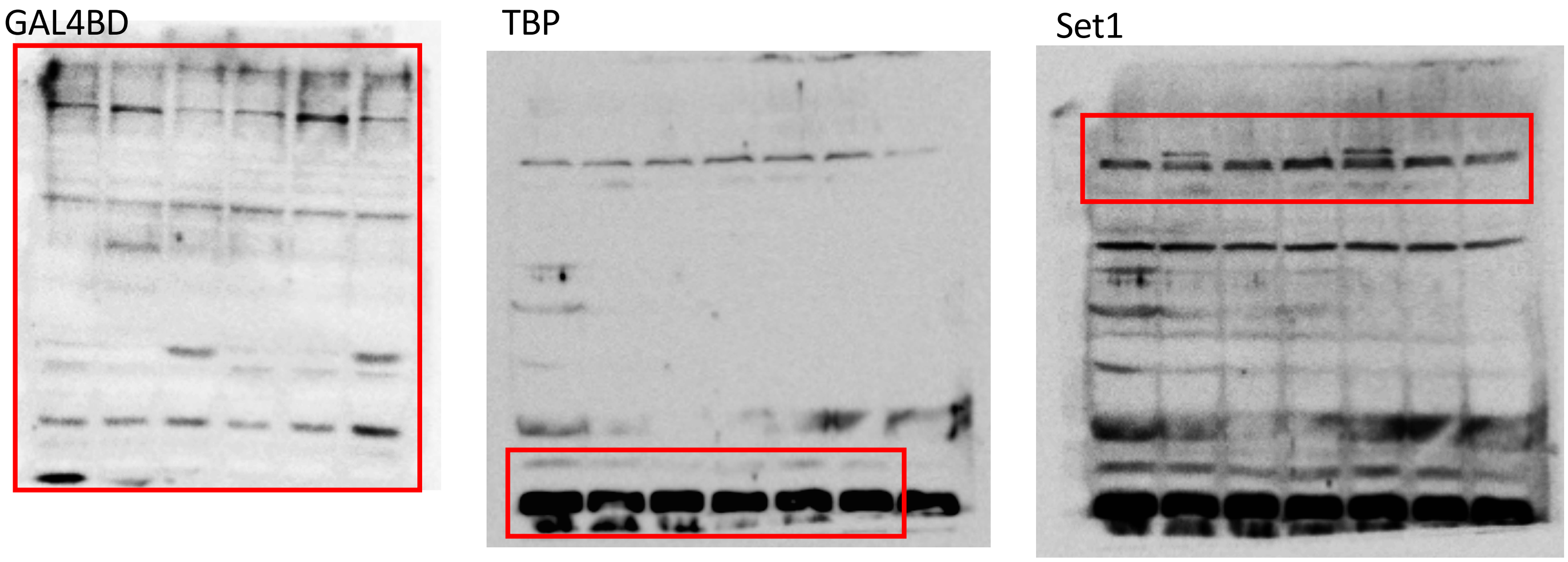

Supplementary Fig. 1d

-Ura, -Leu

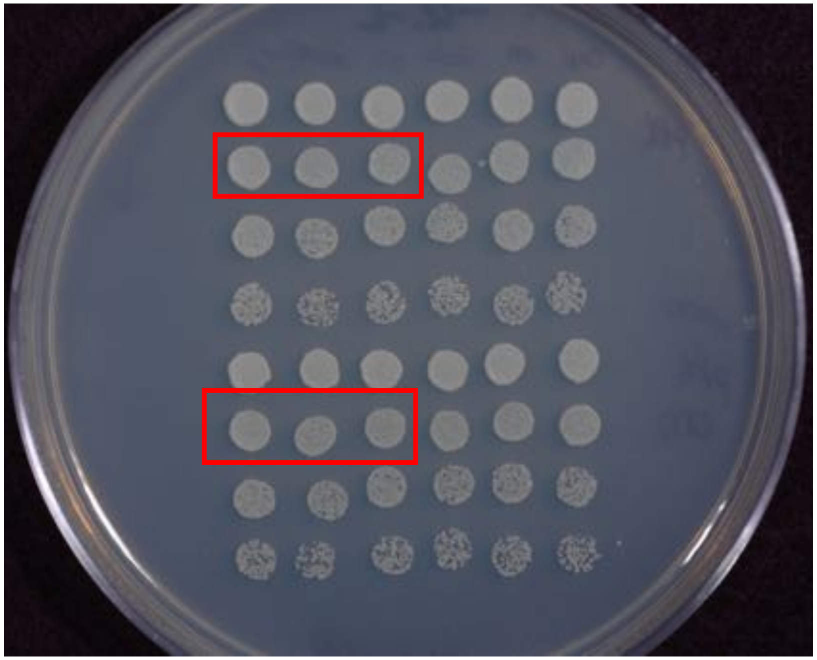

-Ura, -Leu, -His

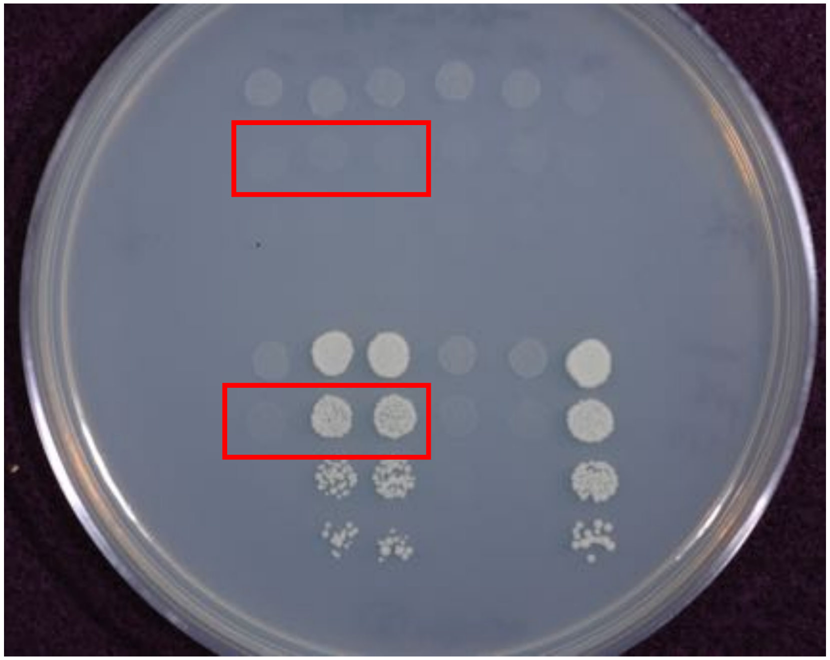

Supplementary Fig. 2a

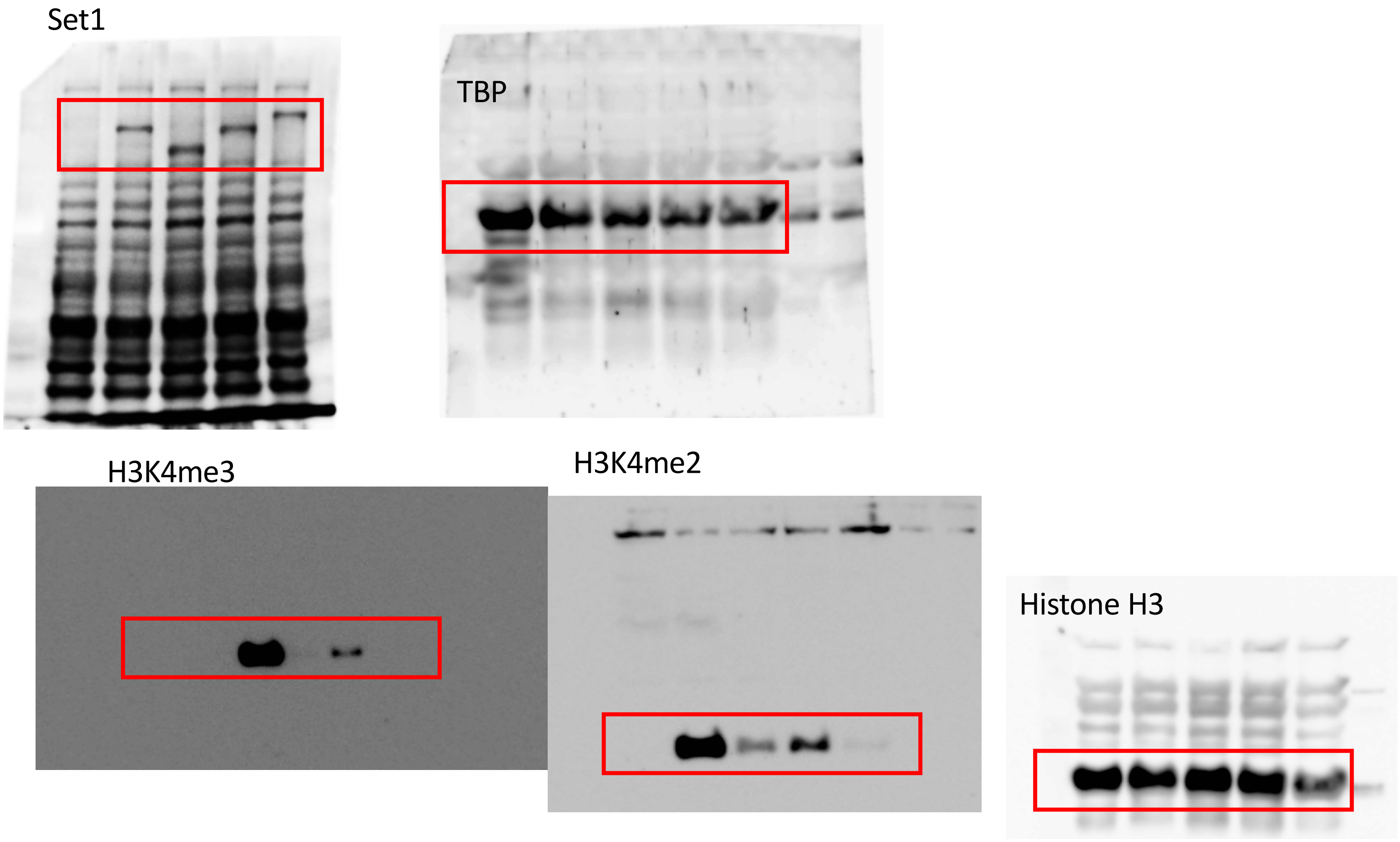

Supplementary Fig. 2b

GAL4BD

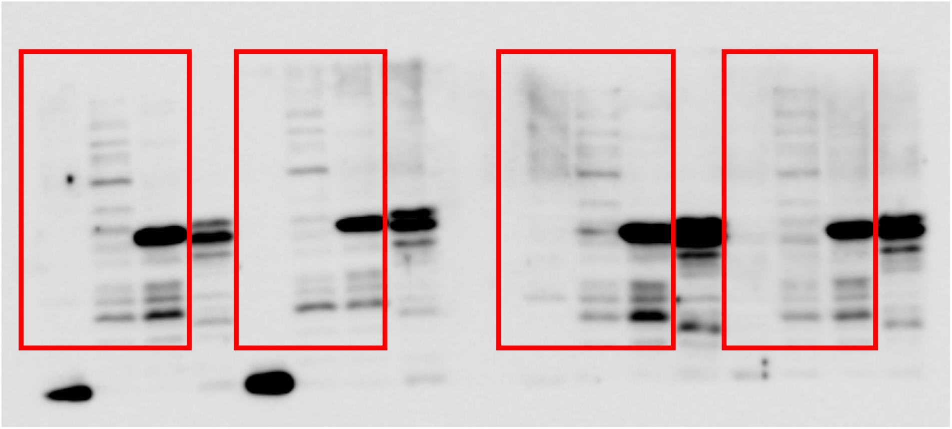

TBP

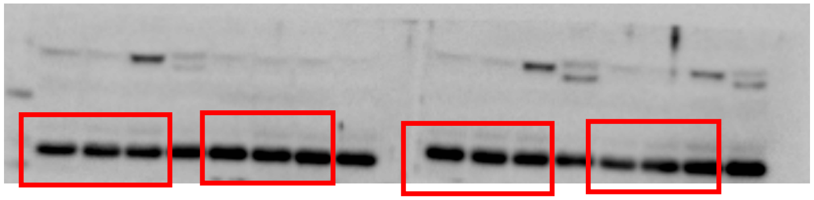

Supplementary Fig. 3a

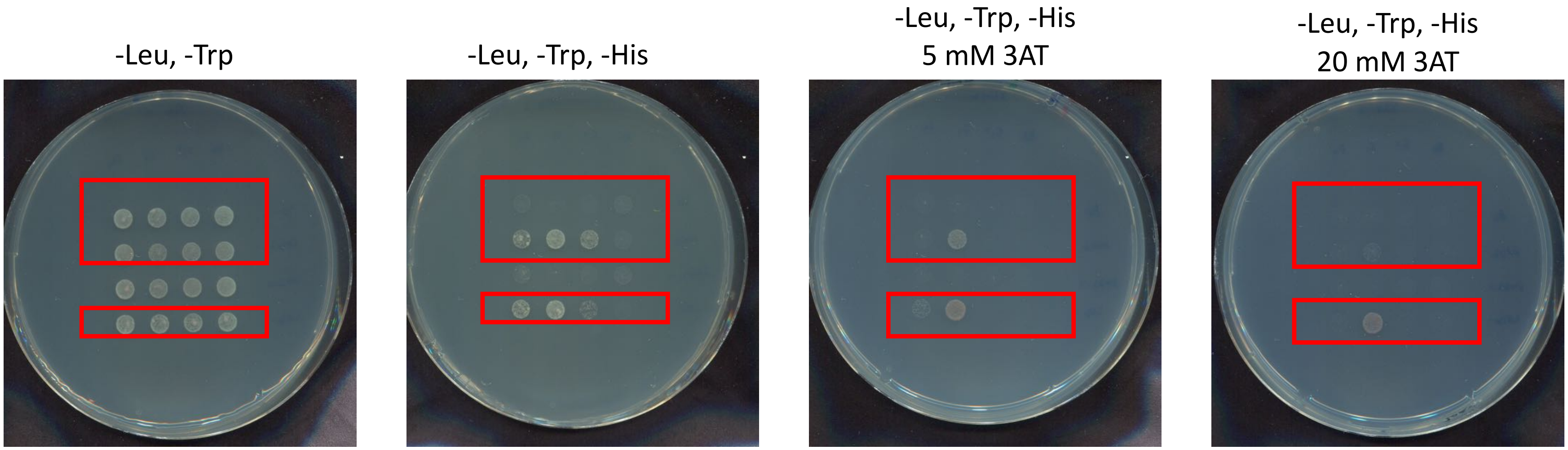

Supplementary Fig. 3b

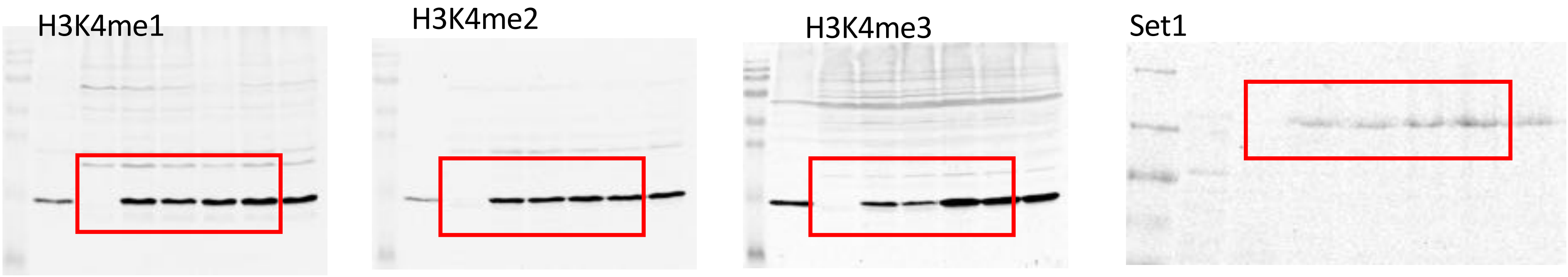

Supplementary Fig. 5c

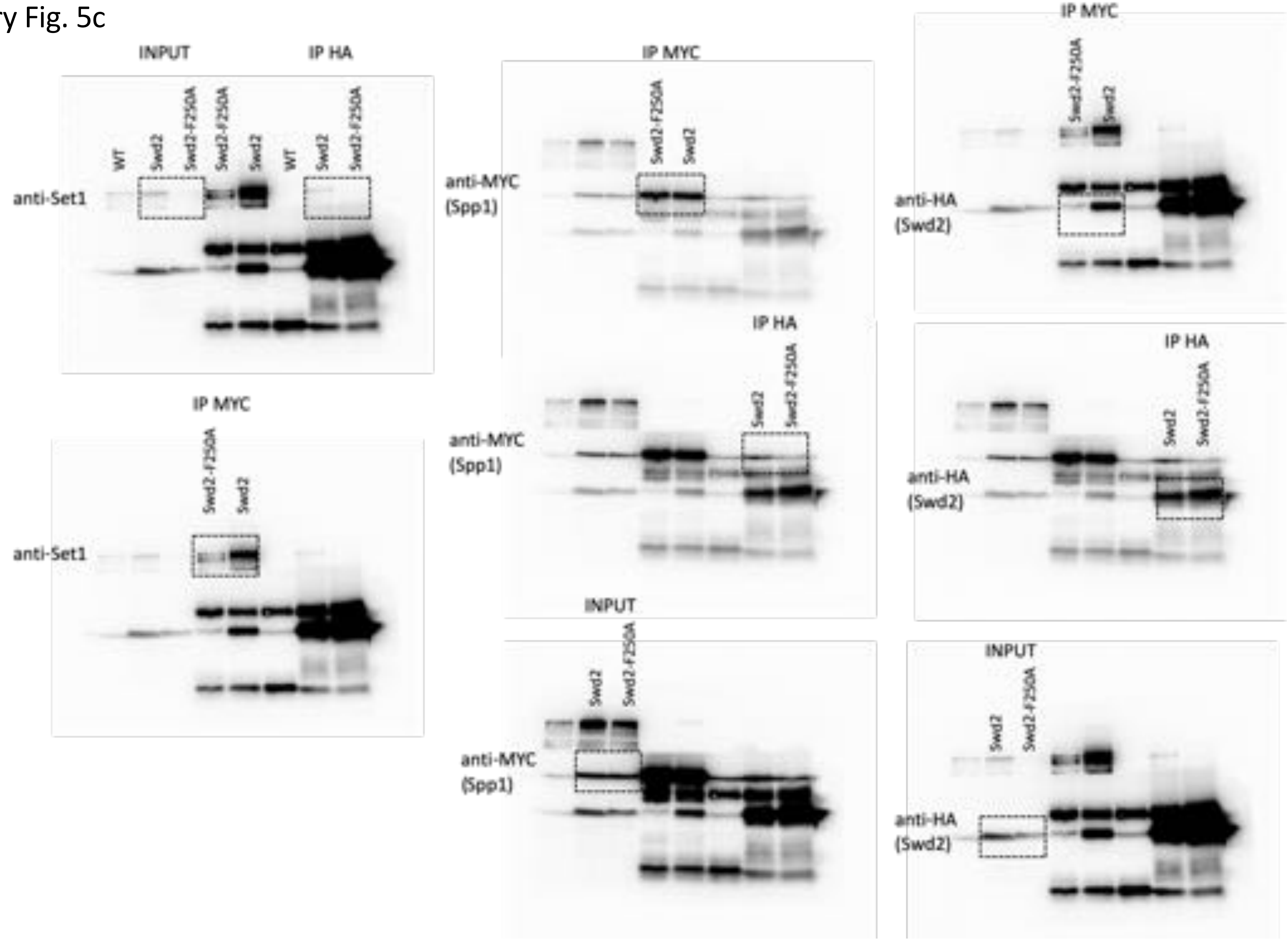

Supplementary Fig. 5c

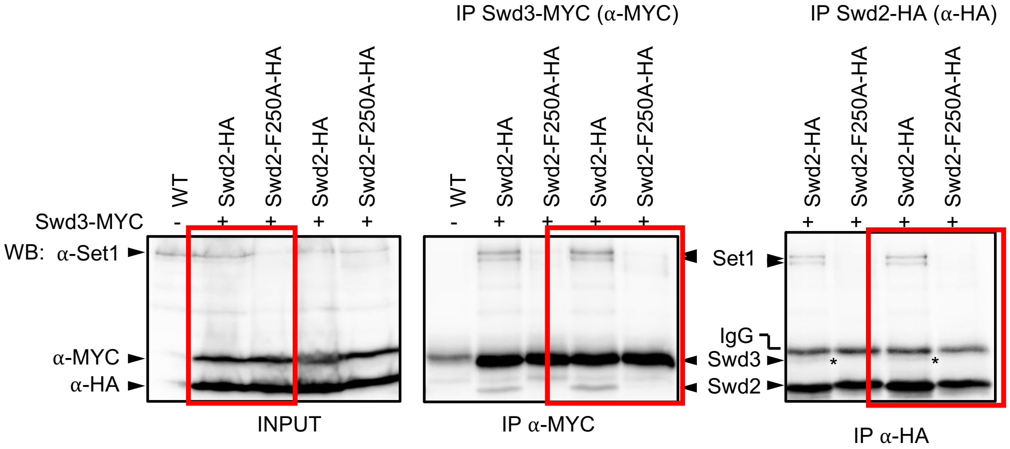

Supplementary Fig. 5d

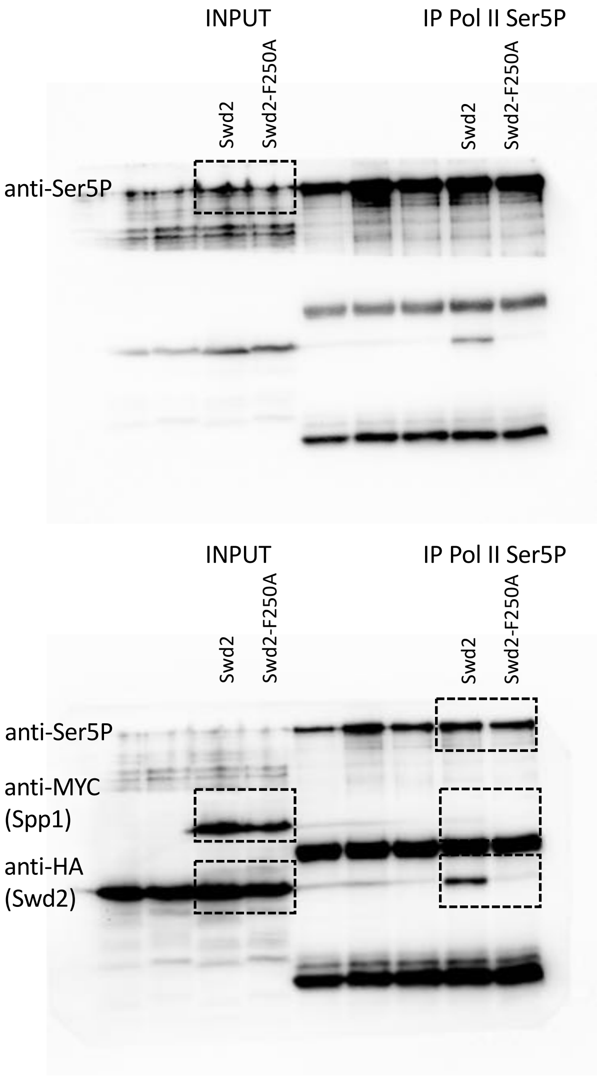

Supplementary Fig. 6a

Set1

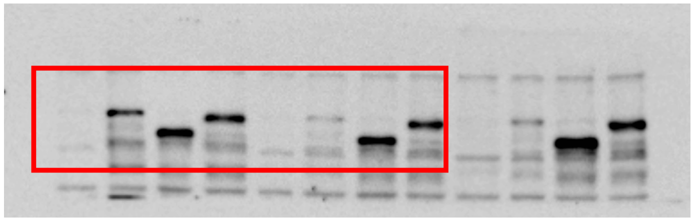

TBP

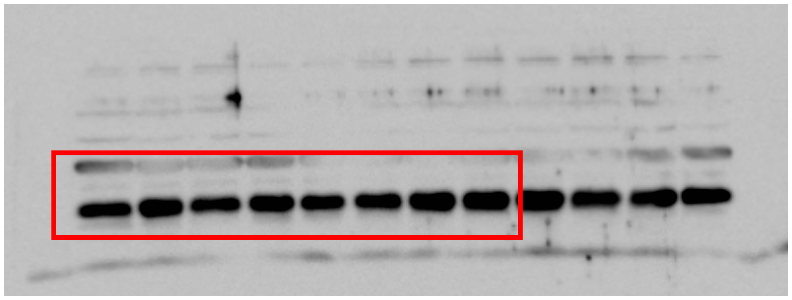

H3K4me3

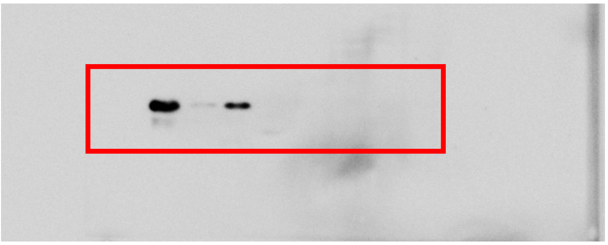

H3K4me2

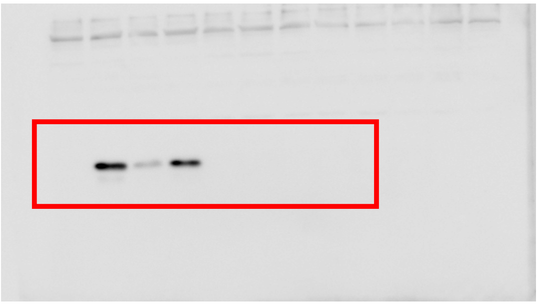

Histone H3

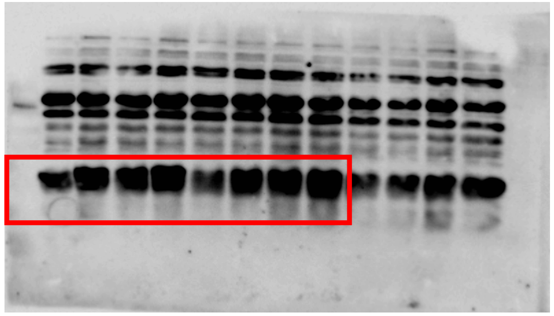

Supplementary Fig. 6b

Set1, FLAG IP

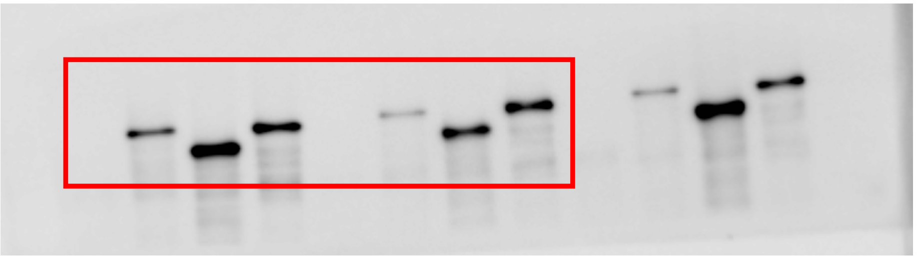

Set1, input

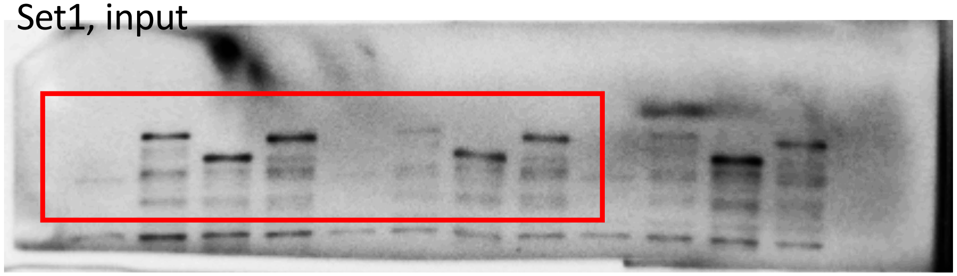

Ser5P, FLAG IP

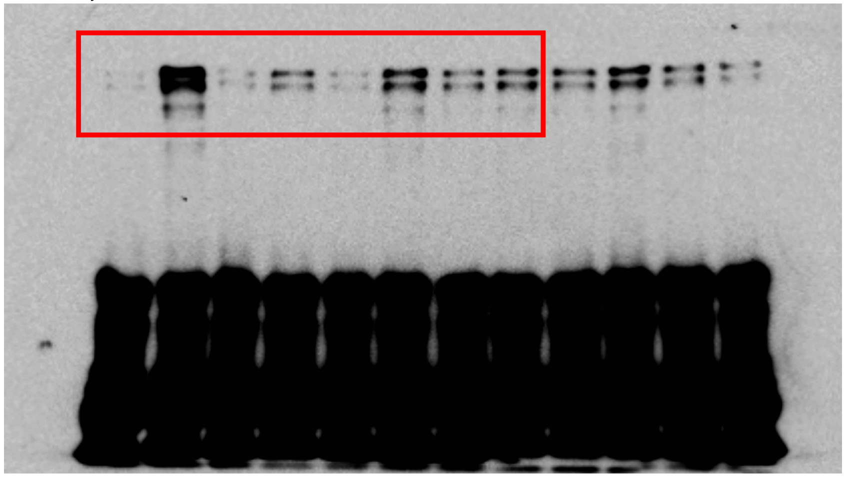

Ser5P, Input

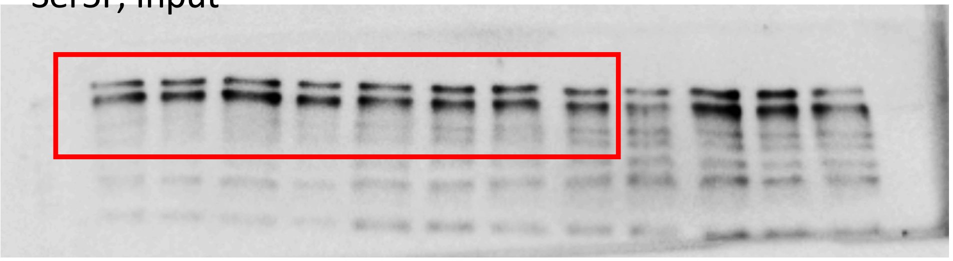

Supplement: Supplementary file 4 — Source Data [file 41467_2020_16082_MOESM4_ESM.pdf]
